# Anilinopyrimidine Resistance in *Botrytis cinerea* Is Linked to Mitochondrial Function

**DOI:** 10.3389/fmicb.2017.02361

**Published:** 2017-11-30

**Authors:** Andreas Mosbach, Dominique Edel, Andrew D. Farmer, Stephanie Widdison, Thierry Barchietto, Robert A. Dietrich, Andy Corran, Gabriel Scalliet

**Affiliations:** ^1^Syngenta Crop Protection AG, Stein, Switzerland; ^2^National Center for Genome Resources, Santa Fe, NM, United States; ^3^Syngenta Biotechnology Inc., Research Triangle Park, NC, United States; ^4^Syngenta Jealott's Hill International Research Centre, Bracknell, United Kingdom; ^5^BIOtransfer, Montreuil, France

**Keywords:** anilinopyrimidine, fungicide resistance, *Botrytis cinerea*, gray mold, mitochondria, ABC transporter, NADH kinase

## Abstract

Crop protection anilinopyrimidine (AP) fungicides were introduced more than 20 years ago for the control of a range of diseases caused by ascomycete plant pathogens, and in particular for the control of gray mold caused by *Botrytis cinerea*. Although early mode of action studies suggested an inhibition of methionine biosynthesis, the molecular target of this class of fungicides was never fully clarified. Despite AP-specific resistance having been described in *B*. *cinerea* field isolates and in multiple other targeted species, the underlying resistance mechanisms were unknown. It was therefore expected that the genetic characterization of resistance mechanisms would permit the identification of the molecular target of these fungicides. In order to explore the widest range of possible resistance mechanisms, AP-resistant *B*. *cinerea* UV laboratory mutants were generated and the mutations conferring resistance were determined by combining whole-genome sequencing and reverse genetics. Genetic mapping from a cross between a resistant field isolate and a sensitive reference isolate was used in parallel and led to the identification of an additional molecular determinant not found from the characterized UV mutant collection. Together, these two approaches enabled the characterization of an unrivaled diversity of resistance mechanisms. In total, we report the elucidation of resistance-conferring mutations within nine individual genes, two of which are responsible for almost all instances of AP resistance in the field. All identified resistance-conferring genes encode proteins that are involved in mitochondrial processes, suggesting that APs primarily target the mitochondria. The functions of these genes and their possible interactions are discussed in the context of the potential mode of action for this important class of fungicides.

## Introduction

Anilinopyrimidine (AP) fungicides were introduced into the crop protection market between 1992 and 1995. In total, the three closely related molecules pyrimethanil (Neumann et al., 1992), cyprodinil (Heye et al., 1994), and mepanipyrim (Maeno et al., 1990) were launched for the control of a range of diseases caused by ascomycetes. In practice, AP fungicides are mostly used for the control of pathogens of fruits and vegetables, and especially for the control of gray mold (*Botrytis cinerea*) on fruits (grape, berries) vegetables (tomato, pea) and ornamentals (rose), and for the control of apple scab (*Venturia inaequalis*) (Müller et al., 1998). In addition, cyprodinil-containing products are used for the control of cereal diseases such as powdery mildew (*Blumeria graminis* f.sp *tritici*) and *Parastagonospora nodorum* on wheat, and eyespot, powdery mildew, net blotch, and scald on barley caused by *Oculimacula* spp., *Pyrenophora teres* and *Rhynchosporium commune*, respectively. Pyrimethanil-containing products are used for the control of *Ascochyta* spp. in legumes, *Mycosphaerella* spp. in banana, pea and other vegetables, and for post-harvest diseases (e.g., *Aspergillus* and *Penicillium* spp.).

AP fungicides are currently classified as potential methionine biosynthesis inhibitors (FRAC^1^ target code D1). This classification emerged from early mode of action studies mainly performed on *B*. *cinerea*, which displayed partial reversal of growth inhibition when sulfur-containing amino acids, and in particular methionine or its upstream metabolite homocysteine, were added to minimal culture media containing the fungicide (Leroux, 1994; Masner et al., 1994; Leroux et al., 1995). Analysis of sulfur assimilation from AP-treated mycelium in the presence of [^35^S] disodium sulfate as a sulfur source displayed a slight accumulation of [^35^S] cystathionine, concomitant with a 3-fold reduction of [^35^S] methionine (Fritz et al., 1997). The lack of reversal by cystathionine, a metabolite one step before homocysteine, suggested the mode of action could be methionine biosynthesis inhibition through the inhibition of cystathionine β-lyase (Masner et al., 1994; Fritz et al., 1997). However, enzymatic studies using *B*. *cinerea* protein extracts revealed no or very minor inhibition of cystathionine β-lyase at high doses of anilinopyrimidines (Sierotzki et al., 2001; Fritz et al., 2003). An analysis of the cystathionine β-lyase and cystathionine γ-synthase-encoding genes in resistant and sensitive isolates also showed no link between the phenotypes and the genotypes for these loci (Sierotzki et al., 2001; Fritz et al., 2003). Finally, methionine and homocysteine reversal studies performed with *Penicillium digitatum* failed to show any reversal effect, suggesting that the metabolite-chemical interaction observed in *Botrytis* may be due to an indirect species-specific effect (Kanetis et al., 2008).

Another important feature of the AP mode of action was highlighted from other studies performed on *B*. *cinerea* with mepanipyrim and pyrimethanil (Miura et al., 1994; Milling and Richardson, 1995). These studies showed that AP fungicides prevent the secretion of fungal hydrolytic enzymes such as laccases, lipases, proteases, sugar modifying (invertase), and cell wall degrading enzymes (cutinases, pectinases, and cellulases), suggesting that differential sensitivity to AP fungicides on different growth media may be caused by differential requirements for extracellular enzymes necessary for the mobilization of nutrients (Miura et al., 1994; Milling and Richardson, 1995). Altogether, these previous mode of action studies on AP fungicides did not lead to the identification of their molecular target and emphasized the importance of molecular approaches in deciphering the primary target site of these molecules (Leroux et al., 2002).

AP fungicides display class-specific cross-resistance, which is consistent with a similar mode of action in controlled species (Leroux et al., 1999). This fungicide class is considered as being of medium resistance risk by the Fungicide Resistance Action Committee (FRAC). Good performance is still observed in the field with the exception of situations where a high frequency of resistance is present (Forster and Staub, 1996; Hilber and Hilber-Bodmer, 1998; Latorre et al., 2002). However, resistance developed quite rapidly in *B*. *cinerea*, as AP resistance was already detected in a trial site after a few seasons of product application prior to the launch of cyprodinil. This particular feature led to the simultaneous introduction of a fungicide mixture combining two novel active ingredients (cyprodinil and fludioxonil) for a sustained control of *B*. *cinerea* by CibaGeigy (Forster and Staub, 1996; Scalliet et al., 2016). More generally, beyond *Botrytis* control, AP fungicides are frequently mixed with active ingredients of different modes of action to either broaden their biological spectrum or to mitigate against resistance development. Resistant field isolates have been observed in multiple species including *B*. *cinerea* (Hilber and Hilber-Bodmer, 1998; Latorre et al., 2002; Chatzidimopoulos et al., 2013; Leroch et al., 2013), *V. inequalis* (Fiaccadori et al., 2007), *P*. *teres* (Syngenta internal knowledge), *A*. *alternata* (Fairchild et al., 2013; Malandrakis et al., 2015), *Oculimacula* spp. (Leroux et al., 2013), *Penicillium* spp. (Kanetis et al., 2008; Karaoglanidis et al., 2011). However, in most of the cases the presence of resistant isolates at low frequency is not considered to be of practical relevance, in particular when appropriate product usage recommendations are followed (FRAC). Interestingly, within *B*. *cinerea* field populations from grape, the frequency of AP fungicide-resistant isolates appeared to be rather low and stable over the years in comparison to other fungicide classes (Walker et al., 2013). The frequency of resistance heavily depended on the crop and associated spray regime (Leroch et al., 2013; Walker et al., 2013; Scalliet et al., 2016), suggesting resistance encompasses a fitness cost. However, no fitness penalty has been demonstrated so far using either *in vitro* or *in vivo* lab methodologies (Bardas et al., 2008).

Detailed fungicide sensitivity analysis of *B*. *cinerea* field isolates from French vineyards enabled the description of three different AP-shifted phenotypes (Ani^R1−3^) among which only Ani^R1^, displaying the largest sensitivity shift *in vitro* (over 20-fold and up to 250-fold), was shown to have a practical relevance for the efficacy of solo applications of APs in the field (Forster and Staub, 1996; Leroux et al., 1999). Ani^R2^ and Ani^R3^ phenotypes correspond to *in vitro* sensitivity shifts below 20-fold and are caused by the overexpression of different multidrug transporters, which concomitantly affect sensitivity to a range of unrelated fungicides (Leroux et al., 1999). The molecular factors responsible for the Ani^R2^ and Ani^R3^ multidrug resistance (MDR) phenotypes have been characterized recently (Kretschmer et al., 2009; Leroch et al., 2013; Leroux and Walker, 2013). Despite the first description of Ani^R1^ phenotypes dating from the mid-90s (Forster and Staub, 1996; Hilber and Hilber-Bodmer, 1998; Leroux et al., 1999), the underlying molecular mechanism has not yet been elucidated. Segregation studies from sexual crosses between Ani^R1^ and sensitive isolates established a monogenic inheritance, suggesting the involvement of a single resistance-conferring gene (Hilber and Hilber-Bodmer, 1998; Chapeland et al., 1999). Because Ani^R1^ corresponds to strong resistance and is specific to AP fungicides, the identification of the molecular resistance determinant has long been suggested as a primary means to unravel the mode of action of APs (Leroux et al., 2002).

In this paper we took advantage of our contribution to the recent assembly of a gapless *B*. *cinerea* genome (Van Kan et al., 2017) to (i) determine resistance mechanisms in AP-resistant UV lab mutants using a whole-genome sequencing approach, (ii) determine the most frequent resistance mechanism in Ani^R1^ field samples using a sequenced mapping population that we initially generated for producing a high density linkage map of *Botrytis*, and (iii) used these complementary information sets to characterize *B*. *cinerea* field populations. Our work enabled us to demonstrate a partial overlap between the two approaches and to discover an unrivaled level of complexity in terms of resistance mechanisms. We demonstrated the individual involvement of mutations within at least eight separate genes from our UV mutagenesis approach and characterized a wide range of resistance-conferring mutations that occur in two distinct genes in field isolates.

All resistance-conferring mutations affected nuclearly-encoded mitochondrial proteins, supporting a mitochondrial mode of action for AP fungicides. We discuss known and proposed interactions between these genes, present possible explanations for the observed phenotypes and hypothesize on the likely target of this important class of fungicides.

## Materials and methods

### Fungal strains, media, and culture conditions

The fully sequenced anilinopyrimidine (AP) sensitive *B. cinerea* reference strain B05.10 was used as genetic background for UV mutagenesis screening and for all reverse genetic confirmation of cyprodinil (CDL) resistance-conferring mutations. The two AP-resistant field isolates 09Bc11 (Van Kan et al., 2017) and BAR633 (kindly provided by Stefania Pollastro, University of Bari, Italy) were used for genetic crossing experiments. *B. cinerea* field samples, which correspond to either mixed populations of up to three genotypes or single isolates as determined by subsequent genotyping methods, were mostly collected from trial sites and obtained by collection of conidia from the surface of infected plant tissues using sterile cotton swabs. Samples were propagated and phenotypically tested at BIOtransfer laboratory (Montreuil, France). Samples isolated in 2014 originate from grape (*n* = 303, from Austria, Croatia, France, Germany, Hungary, Italy, Portugal, Romania, Slovenia, Spain, and Switzerland) and strawberry (*n* = 40, from Belgium, France, Germany, Italy, Spain, and Sweden), as shown in Table S1 excluding samples displaying multiple genotypes. *B*. *cinerea* samples for which Sanger sequencing was performed are listed in Table S2.

For non-selective propagation of *B*. *cinerea* GEA plates were used (frozen peas 160 g·L^−1^, homogeneously mixed in a blender; sucrose 5 g·L^−1^; agar 20 g·L^−1^). CDL-resistant transformants were selected on SHA (0.6 M sucrose; 5 mM Tris-HCl, pH 6.5; 1 mM (NH_4_)H_2_PO_4_; Bacto agar (Becton, Dickinson and Company, 214030) 7 g·L^−1^), and the subsequent propagation and basic resistance phenotyping assays were done on Vogel's minimal agar (Vogel's salts 50x concentrate, Vogel, 1956 20 mL·L^−1^; sucrose 15 g·L^−1^; agar 15 g·L^−1^), with the respective appropriate CDL supplement. Cultures on agar plates were incubated at 20°C, 16 h white light, and 60% relative humidity, unless stated otherwise. Field samples were purified from bacterial and fungal contaminations at BIOtransfer by suspending the conidia in sterile distilled water supplemented with 50 mg·L^−1^ enrofloxacin and propagated on Acidified S agar plates [sucrose 5 g·L^−1^; malt extract 1 g·L^−1^; Ca(NO_3_)_2_ 1 g·L^−1^; KNO_3_ 250 mg·L^−1^; MgSO_4_ ·7H_2_0 250 mg·L^−1^; KH_2_PO_4_ 125 mg·L^−1^; K_2_HPO_4_ 125 mg·L^−1^; citric acid 1 g·L^−1^ (5 mg·L^−1^ added before, the rest after autoclaving); agar 25 g·L^−1^] at 25°C for 4 days, before transfer to GEA plates for spore production (19°C, 7 days under black light). Mycelium material for protoplast generation was produced in NY liquid medium (yeast extract 200 mg·L^−1^; malt extract 20 g·L^−1^; pH 5.5). GG medium (KH_2_PO_4_ 1.5 g·L^−1^; MgSO_4_ ·7H_2_O 750 mg·L^−1^; gelatin from porcine skin 4 g·L^−1^; glucose 4 g·L^−1^) was used for liquid culture dose-response tests.

### Oligonucleotides, PCRs, and pyrosequencing assays

All oligonucleotides were purchased from Microsynth AG (Balgach, Switzerland). Genomic DNA used as template for polymerase chain reactions was prepared using the MagAttract 96 DNA Plant Core Kit (QIAGEN, 67163), based on freeze-dried sporulating mycelium of the respective field samples, crossing progeny isolates (both harvested from GEA plates), UV mutants, or transformants (both from Vogel's minimal agar with the respective appropriate CDL supplement). Each PCR was performed according to the conditions recommended by the respective manufacturer of the polymerase, and using ~50 mg of DNA template per reaction. PCR primers used to amplify sequences for Sanger sequencing or reverse genetic experiments (transformations) are listed in Table S3. PCR products for reverse genetic validation of resistance-related mutations and for Sanger sequencing were amplified with Thermo Scientific Phusion Hot Start II High-Fidelity DNA Polymerase (ThermoFisher Scientific, F549L). For the long products required to map the *Bcpos5* locus, LongAmp Taq DNA Polymerase (NEB, M0323S) was used. DNA Sanger sequencing was done at Microsynth AG or internally (Applied Biosystems 3130 Genetic Analyzer).

Oligonucleotides designed for pyrosequencing assays, together with other assay-related information, are shown in Table S4. PCR products for pyrosequencing assays were amplified from DNA templates of field samples (of which some represented mixed populations) or crossing progeny isolates with GoTaq® G2 Hot Start Polymerase (Promega, M7405). Pyrosequencing was performed on a PyroMark Q96 ID (Biotage/QIAGEN), according to the protocols provided by the manufacturer (Allele Quantification and SNP Genotyping assays in the PyroMark Assay Design software v2.0).

### *In vitro* fungicide sensitivity tests of monitoring field samples and single strain cross-resistance assays

Chemicals used for *in vitro* fungicide sensitivity and cross-resistance assays were purchased from Sigma-Aldrich: Cyprodinil (CDL, 34389), fludioxonil (FDL, 46102), pyrimethanil (31577), mepanipyrim (33970). Stock solutions of antifungal compounds were prepared at 10 g·L^−1^ in dimethyl sulfoxide (DMSO). Sensitivity assays of the original *B*. *cinerea* monitoring field samples (Table S1), and of the mapping population from the cross between 09Bc11 and BAR633 (see section Mapping of Resistance Loci in Field Isolates and Phenotypic Characterization), were performed at BIOtransfer: Conidia were picked with an agar plug from sporulating cultures on GEA and suspended in sterile distilled water. The spore suspension was then adjusted to 2·10^5^ conidia per ml in GG medium. Technical active ingredients dissolved in DMSO were used to prepare adapted concentration ranges in sterile distilled water. Each well of a 96-well microtiter plate was filled with 100 μL of 2x concentrated fungicide solution and 100 μL of conidia suspension. The final concentration of DMSO in each well including the solvent control was 1% (v/v). Final dose ranges used for monitoring samples were 0, 0.006, 0.032, 0.16, 0.8, 4, 20, 100 mg·L^−1^ for CDL and 0, 0.003, 0.008, 0.021, 0.128, 0.8, 2, 5 mg·L^−1^ for FDL. Concentrations used for phenotyping the 09Bc11 x BAR633 mapping population ranged between 0.0017 and 100 mg·L^−1^ with 1:3x dilution steps. For all compounds and concentrations, each *B*. *cinerea* sample or progeny isolate was tested in technical duplicate. Microtiter plates were incubated in the dark at 19°C for 3 days, before measuring optical density at 450 nm with a Biotek plate reader (ELX800 UV). Measured values were subtracted from the blanks measured directly after inoculation. EC_50_ values were calculated using the Grafit 5 software (Erythacus Ltd.).

Liquid culture cross-resistance assays were performed using *B*. *cinerea* conidia of B05.10, single spore isolates based on the original field samples listed in Tables S1, S2, or transformants (see section Validation of Resistance-Related Mutations by Homologous Recombination). Spores were harvested from 7 to 10 days old cultures on GEA. The spores were suspended in GG medium and adjusted to a density of 10^6^ conidia per mL. The suspensions were then diluted 1:10x with GG medium, and 198 μL were distributed into each well of a 96-well microtiter plate (Costar 3370, Corning), already containing 2 μL of 100x concentrated compound solutions in DMSO, resulting in a final DMSO concentration in all assays of 1% (v/v). Dilution series of compounds were prepared in ten 1:3x dilution steps, starting from 10 g·L^−1^ stock solutions, resulting in a final concentration range from 0.0017 to 100 mg·L^−1^, plus a solvent control. Optical density of the cultures at 600 nm was measured directly after treatment (blank) and after 3 days of incubation in a growth chamber at 20°C in the dark, using the EnSpire 2300 Multilabel Reader (PerkinElmer). EC_50_ values were calculated using GraphPad Prism 6.0 (GraphPad Software, USA), with the function: Dose-response–inhibition, log(inhibitor) vs. response–Variable slope (four parameters).

### UV mutagenesis and phenotypic characterization

Conidia of *B*. *cinerea* B05.10 were collected from 7 to 12 days old cultures on GEA. Either 1·10^6^, 5·10^6^, or 2·10^7^ spores were suspended in sterile water and spread onto Vogel's agar 9 cm Petri dishes supplemented with CDL. The selective concentrations of CDL varied between 10 and 100 mg·L^−1^. For a detailed description of the different conditions applied see Table S5. UV mutagenesis was performed using 65 mJ·cm^−2^ treatment with a UV Stratalinker 2400 (Stratagene). Non-selective agar plates as well as non-UV-treated controls were included. After treatment the plates were incubated for at least 10 days in the dark at 20°C. Single colonies were transferred to new selective Vogel's agar plates with a similar concentration of CDL for secondary selection and left to sporulate under light. Isolates were phenotypically tested and only those showing stable resistance were used for further experiments (Table S5).

### Genotypic characterization of UV mutants by next generation sequencing

Thirteen resistant UV mutants and the sensitive B05.10 wild type reference were selected for whole-genome sequencing. Illumina sequencing library preps were generated as described (Van Kan et al., 2017). Sequencing was done on an Illumina HiSeq 2000 (Illumina Inc., San Diego, California, USA) as either 54 or 100 cycle paired end runs, with an average yield of 3.4 Gb per sample (range 1.4–5.4 Gb per sample).

Paired end Illumina reads were aligned to the reference genome assembly described (Van Kan et al., 2017), using the version of the GSNAP aligner released on 2013-03-31 requiring at least 94% identity to the reference (penalizing indels with a score of three relative to a mismatch), and excluding alignments requiring hard clipping. Uniquely aligning reads were used to call variants using the Alpheus pipeline (Miller et al., 2008) with initial filtering parameters set at a minimum of 2 reads within a sample calling the variant with minimum average base quality of 10 and requiring at least 20% of the reads in the UV mutant sample to support the variant call. Variants were annotated with respect to effects on protein coding regions and then further filtered by requiring at least 10 reads of unique coverage, a variant frequency of ≥70% in the UV mutant and of ≤10% in the reference strain. Candidate mutations were assessed from the resulting subset of SNPs and small indels. Table S6 lists the non-synonymous candidate mutations and the total number of high-confidence mutations obtained per UV isolate.

### Mapping of resistance loci in field isolates and phenotypic characterization

The cross between 09Bc11 (Ani^R1^ phenotype) and B05.10, and the generation of a genetic linkage map for 70 progeny isolates (35 sensitive and 35 resistant toward CDL) have been described previously (Van Kan et al., 2017). A second cross was performed between 09Bc11 (maternal) and strain BAR633 (Ani^R1^) according to established procedure (Faretra et al., 1988). EC_50_ values were determined at BIOtransfer for 169 progeny isolates using the liquid culture sensitivity test described above. Pyrosequencing of individual progeny isolates was used to genotype for the presence or absence of resistance-conferring mutations within the *Bcpos5* and *Bcmdl1* genes (pyrosequencing assays Bcpos5-AQ-L412F and Bcmdl1-AQ-E407K, see Table S4).

### Validation of resistance-related mutations by homologous recombination

Genomic regions encompassing the identified SNPs suspected to confer AP resistance were amplified from genomic DNA by PCR, using Phusion Hot Start II High-Fidelity DNA Polymerase (ThermoFisher Scientific, F549L) (primers listed in Table S3), including flanking regions of at least 500 bp up- and downstream of the respective SNPs to facilitate efficient homologous integration at the target loci. To prevent the potential selection of ectopically integrated functional copies of the genes, primers were designed to omit either the 5′ or 3′ or both ends of the coding sequences. However, for particularly short genes (*BcoliC* and *Bcmix17*) full length products of the coding sequences were transformed because of the requirement of having sufficiently long flanking regions. Complete amplicons of coding sequences were also used if previous transformation attempts with truncated sequences did not yield resistant colonies. To prevent the identification of false positive mutations, wild type (B05.10) amplicons of the respective sequences were transformed as negative controls. *Bcpos5* and *Bcmdl1* alleles from Ani^R1^ field samples were amplified from the following sources: *Bcpos5*^V273I^: 10-Bc-187, *Bcpos5*^P293S^: 14-Bc-004, *Bcpos5*^P319A^: 14-Bc-105, *Bcpos5*^G408R^: 14-BC-183^s^, *Bcpos5*^G408V^: 14-BC-056, *Bcpos5*^L412F^: 09Bc11, *Bcpos5*^L412V^: 14-BC-007^s^, *Bcmdl1*^E407K^: 14-BC-020^s^, *Bcmdl1*^S466R^: 10-BC-041^s^ (see Table S2).

In order to narrow the Ani^R1^ mapping window of cross 09Bc11 x B05.10 (see previous section Mapping of Resistance Loci in Field Isolates and Phenotypic Characterization) and identify the genetic factor conferring the resistance phenotype, 10 overlapping fragments of ~11–12 kb length from genomic DNA of the resistant parent 09Bc11, and of the sensitive parent B05.10 as negative control, were amplified by PCR and transformed into B05.10 as described below (primers listed in Table S3). For this approach LongAmp Taq DNA Polymerase (NEB, M0323S) was used. Based on the sole fragment amplified from 09Bc11 that conferred resistance, the same strategy was reiterated using smaller overlapping fragments of ~3.6 kb (Table S3).

Prior to transformation PCR products were purified using the NucleoSpin® Gel and PCR Clean-up kit (Macherey-Nagel), using between 2 and 8 pmoles (6–16 μg) of DNA for transformation. The method for PEG-based transformation of *B*. *cinerea* protoplasts was adapted from a protocol developed for *Fusarium oxysporum* (Malardier et al., 1989). In brief, NY medium in round-bottom flasks was inoculated with 10^8^ conidia of B05.10 per 100 ml and shaken for 16 h at 180 rpm, 20°C. The cultures were centrifuged for 10 min at 3,700 *g*, and the germlings were washed twice in KCl/NaP buffer (545 mM KCl, 91 mM sodium-phosphate; pH 5.8). Ca. 3.5–4 g germlings (wet weight) were re-suspended in 40 ml KCl/NaP buffer, pre-mixed with 600 μL 1 M KOH, and 0.5 g Lysing Enzymes from *Trichoderma harzianum* (Sigma, L1412) and incubated at 70 rpm, 20°C. After 2.5 h protoplasts were filtered through 25 μm nylon meshes, centrifuged for 5 min at 4°C, 2,000 *g*, and washed in ice-cold TMS buffer (1 M sorbitol; 10 mM MOPS, pH 6.3). Protoplasts were then re-suspended in ice-cold TMSC buffer (TMS plus 40 mM CaCl_2_) and the concentration (hemocytometer) adjusted to 2·10^7^ protoplasts per 100 μL. The DNA to be transformed was prepared in 100 μL aliquots in TE buffer supplemented with 40 mM CaCl_2_, and mixed with 100 μL protoplast suspension. After an incubation on ice for 20 min, 160 μL of PEG6000/MS solution (1.2 g PEG6000 in 800 μL MS: 0.6 M sorbitol; 10 mM MOPS, pH 6.3) was added and carefully mixed with the protoplasts. After another 15 min incubation at room temperature, 700 μL TMSC buffer was added and the mixture was centrifuged for 5 min at 1,000 *g*. The protoplasts were re-suspended in 200 μL TMSC and added to 200 mL SHA (42°C), containing CDL (10 mg·L^−1^), and 20 mL aliquots poured into Petri dishes. The plates were incubated at 20°C in the dark for up to 3 weeks and examined for the emergence of resistant colonies, which were picked and further propagated on Vogel's minimal agar with CDL (10 mg·L^−1^). Homologous recombination of the transformed truncated or full-length gene copies within putative transformants was subsequently tested by amplification of the genomic regions encompassing the homologous recombination points (for PCR primers see Table S3), followed by Sanger sequencing.

## Results

### Generation and phenotyping of UV mutants

**Figure 1:**
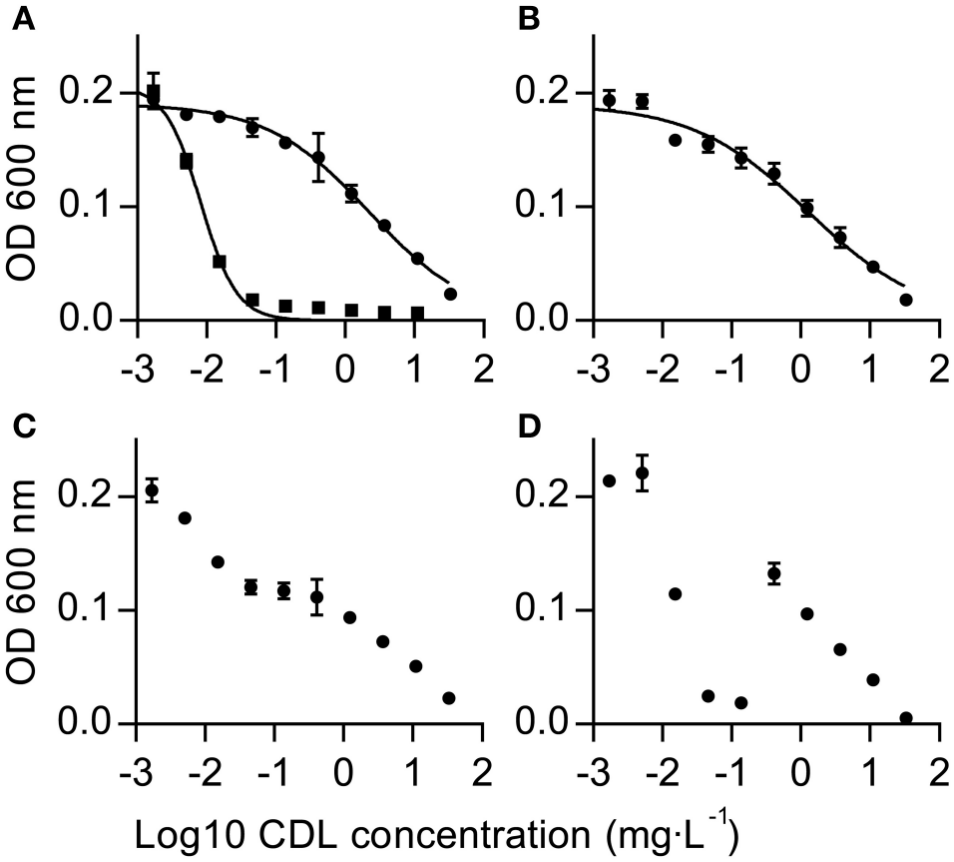
Types of dose-response curves of CDL-resistant UV mutants observed in liquid culture tests. The graphs show mean values of triplicate measurements ± standard deviation. **(A)** Classical monophasic dose-response curves of a CDL sensitive strain (squares) and a resistant UV isolate (circles). **(B)** Very slightly biphasic curve (based on visual ranking). **(C)** Biphasic dose-response (no regression curve). **(D)** “Displaced” biphasic dose-response (no regression curve).

In order to facilitate the identification of anilinopyrimidine (AP) resistance-conferring mutations, UV mutants were generated *in vitro* using the reference *B. cinerea* strain B05.10, for which a well-annotated genome sequence is available (Van Kan et al., 2017). Primary selection was performed on four different concentrations of cyprodinil (CDL), ranging from 10 to 100 mg·L^−1^ (see Materials and Methods). Very rare spontaneous colonies occurred in the non-UV-treated controls (five in total, see Table S5) and were not processed further. Of the 114 original UV isolates, only 66 displayed stable resistance after sub-culturing on selective medium. Isolates within this group showed one out of four distinguishable dose-response phenotypes in liquid culture sensitivity tests: Monophasic dose-response curves (Figure 1A), slightly biphasic dose-response curves (Figure 1B), biphasic dose-response (Figure 1C) and striking “displaced” biphasic dose-responses, as exemplified in Figure 1D. An accurate determination of EC_50_ values for the latter two types of curves was not possible.

### Sequencing of UV mutants and identification of resistance-related SNPs

A collection of 13 UV mutants displaying stable resistance, and covering each of the four distinct dose-response phenotypes, was selected for whole-genome sequencing, together with the non-mutagenized reference isolate. Comparison of the sequencing data with the annotated B05.10 reference genome (see Materials and Methods) revealed single nucleotide polymorphisms (SNPs) and short deletions in the genomes of the mutants that were not found in the non-mutagenized control. By filtering the results at high stringency, up to five non-synonymous SNPs within different predicted genes were identified per UV isolate (Table S6), with little overlap between the isolates. No such mutations were found in strain CDL10-11, although displaying a stable loss of CDL sensitivity. In an attempt to reveal non-synonymous variations occurring at a lower frequency further analysis of the sequencing data of CDL10-11 was undertaken. This led to the identification of premature stop codons within multiple genes including Bcin03g01010, an ortholog of yeast *pet9*, which encodes the mitochondrial inner membrane ADP/ATP carrier. The SNP within this gene was only detected in 46% of the sequencing reads (data not shown).

Assuming that resistance was caused by a single mutation in each of the isolates, the different candidate genes (Table S6) were validated separately using a reverse genetic approach. For transformations, PCR products encompassing the mutated loci were amplified from genomic DNA of mutants and the non-mutagenized B05.10 reference strain as a negative control (see Materials and Methods). For each analyzed mutant except CDL10-11 a single mutated candidate gene resulted in CDL-resistant transformants (data not shown). The presence of the mutations at the targeted genomic loci was subsequently verified by PCR amplification of the genomic region framing the homologous recombination points, followed by Sanger sequencing. In summary, eight different CDL resistance-related genes were identified (Table 1). Mutations in four of them, *Bcdnm1, Bcafg3, Bcphb2*, and *Bcmdl1*, were found to confer resistance even when the transformed PCR products contained coding sequences truncated at either the 5′ or 3′ ends to force homologous integration events. The other four required full length gene copies either because of their small size (*Bcmix17* and *BcoliC*) or because previous transformation attempts with truncated gene fragments failed for unknown reasons (*Bcatm1* and *Bcmcr1*). This full length gene approach presents the disadvantage of possible ectopic integrations of additional functional copies into the genome (not tested). The mutation detected at low frequency within strain CDL10-11, resulting in a premature stop codon within Bcin03g01010, was tested in the same way but only as a 5′-truncated gene copy, which did not result in resistant transformants.

**Table 1:** Validated CDL resistance-conferring mutations and sensitivity data of UV mutants.

***B. cinerea* gene ID**	**Trivial name**	**Codon**	**Amino acid change**	**Liquid culture phenotype**	**EC_50_ CDL (mg·L^−1^)**	**EC_50_ FDL (mg·L^−1^)**	**UV mutant isolate**
Bcin01g01830	*Bcmix17*	GGT▸GAT	G79D	Very slightly biphasic	3.56 ± 0.70	0.03 ± 0.01	*CDL10–12*, 10–41, 20–9^*^, 50–24^*^, 100–7^*^
		GGA▸GAA	G83E	Very slightly biphasic	3.13 ± 0.57	0.03 ± 0.01	CDL10–38^*^, 100–6^*^
Bcin02g02630	*Bcdnm1*	GAA▸GGA	E450G	Monophasic^†^	3.69 ± 0.40	0.03 ± 0.00	*CDL50–2*
Bcin03g02170	*Bcafg3*	CTT▸CCT	L305P	Very slightly biphasic^†^	5.38 ± 3.06	0.03 ± 0.01	*CDL10–40*
Bcin07g01710	*Bcphb2*	TTA▸TCA	L153S	Very slightly biphasic^†^	3.58 ± 0.39	0.04 ± 0.01	*CDL100–12*
Bcin10g00060	*Bcmcr1*	GCT▸–CT	A280 frameshift	Displaced biphasic	n. c.	0.03 ± 0.01	*CDL100*–*3*
		GGG▸GG–^‡^	G295 frameshift	Displaced biphasic	n. c.	0.03 ± 0.01	CDL100–1^*^
Bcin10g01500	*BcoliC*	CGT▸TGT	R33C	Very slightly biphasic	3.29 ± 0.96	0.04 ± 0.01	*CDL10–9*, 20–3
Bcin15g00830	*Bcatm1*	GAA▸AAA	E414K	Biphasic	n. c.	0.03 ± 0.01	*CDL100–5*, 100–11
Bcin16g00820	*Bcmdl1*	GAA▸AAA	E407K	Monophasic	2.84 ± 0.44	0.03 ± 0.01	*CDL10–34*
		GGC▸CGC	G422R	Very slightly biphasic^†^	3.37 ± 0.58	0.03 ± 0.01	*CDL50–8*

*EC_50_ values are the mean of biological triplicate measurements ± standard deviation; and the corresponding isolate names are italicized. Values determined in the same experiment for the reference strain B05.10 were 0.02 ± 0.00 mg·L^−1^ for CDL and 0.03 ± 0.01 mg·L^−1^ for fludioxonil (FDL). B. cinerea trivial names were chosen based on sequence homology to respective genes in yeast. N. c.: Not calculated because of biphasic or “displaced” biphasic dose-response curves*.

* *Not whole-genome sequenced*.

† *Validity of liquid culture phenotype (based on visual ranking) limited to one available UV mutant and mutation not observed in field samples*.

‡ *Not validated by reverse genetics*.

For two of the genes (*Bcmix17* and *Bcmcr1*) further mutations affecting different codons were identified by Sanger sequencing of the coding sequences of mutants that were not selected for whole-genome sequencing (Table 1). The *Bcmcr1* frameshift deletion identified in isolate CDL100-1 was different from that found before in CDL100-3, but both lead to a similar C-terminal extension of the predicted BcMcr1 protein sequence (Figure S1). Furthermore, both isolates displayed similar unusual “displaced” biphasic dose-response curves in liquid culture tests that were not observed for any other resistance-related mutation. Representative dose-response curves for all mutations identified in the UV screening are shown in Figure S2. The resistance factors relative to the sensitive reference strain did not differ much between the UV mutants listed in Table 1, they ranged between 142 and 269-fold for *Bcmdl1*^E407K^ and *Bcafg3*^L305P^, respectively.

### Mapping of a major resistance factor from a field isolate

**Figure 2:**
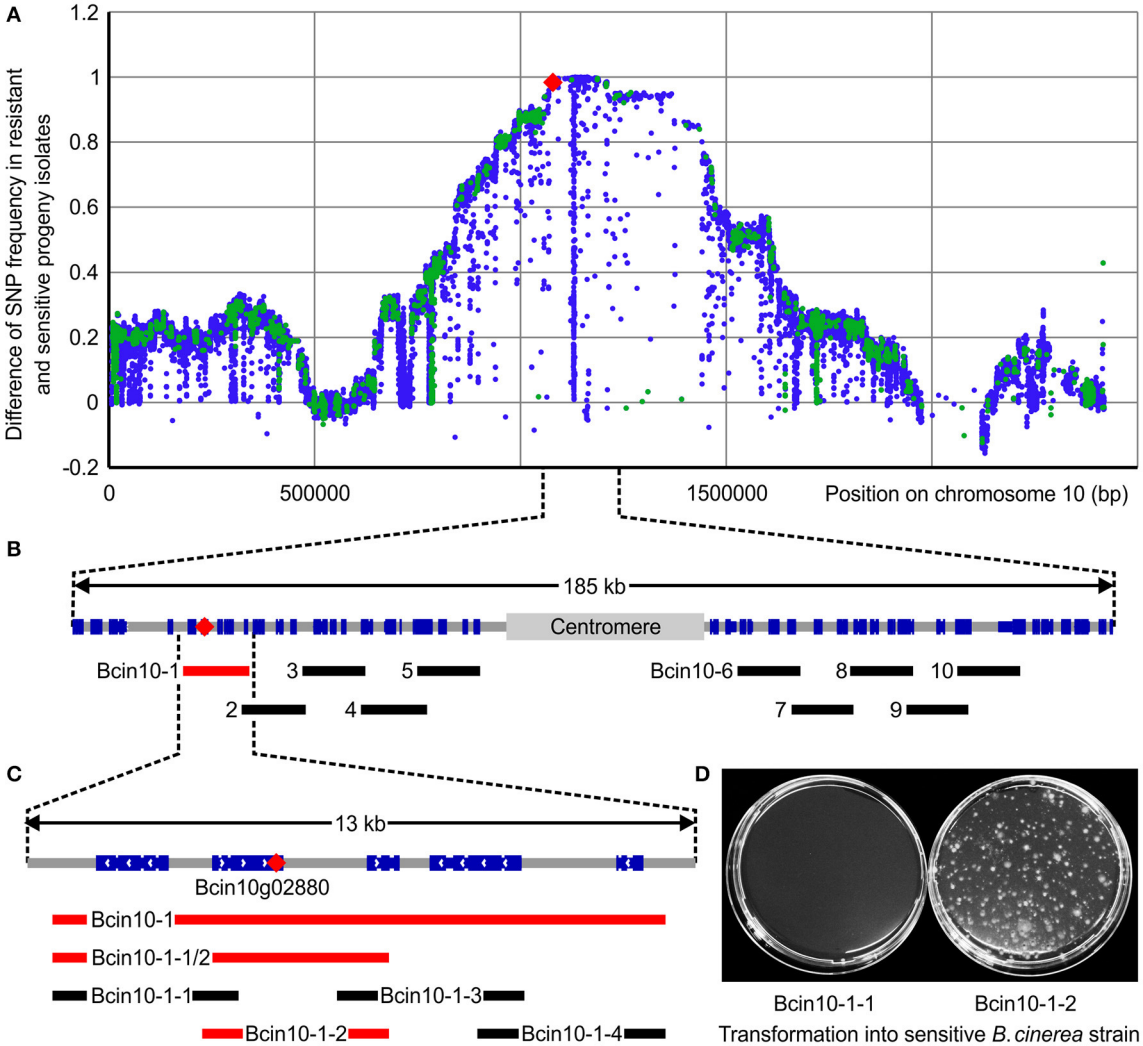
*In silico* bulked segregant analysis mapping and reverse genetic validation of a major gene conferring resistance toward APs in field populations of *B*. *cinerea*. **(A)** Difference in SNP frequencies found in the respective AP-resistant (R) and sensitive (S) bulks plotted against the sequence assembly coordinates of chromosome 10. Frequencies were calculated for each position in the R and S bulks using the formula: Total number of reads representing the SNP at position/total number of reads covering the position in the bulk. The y value for any SNP position can vary between zero for unlinked positions to +1 for positively linked positions (SNP linked to resistance) or to −1 in the case of a negative linkage. Blue dots represent synonymous SNPs or SNPs outside of coding sequences and green dots represent non-synonymous SNPs. The SNP that confers resistance is marked by a red diamond, also in the subsequent sections. **(B,C)** Lower panel boxes B and C represent sections (Chromosome 10: 1,055,000–1,240,000 and 1,074,000–1,087,000, respectively) of the mapping window, with coding sequences of genes marked in blue (exported from IGV; Thorvaldsdottir et al., 2013). PCR products used to identify the R locus by a reverse genetic approach are represented by black or red bars, where red bars indicate fragments conferring R when transformed into the S strain B05.10. **(D)** Reverse genetic validation transformation with genomic fragments Bcin10-1-1 and Bcin10-1-2 (amplified from 09Bc11) transformed into B05.10 and selection on CDL-containing agar. The latter fragment contains the resistance-conferring mutation in gene Bcin10g02880, resulting in resistant colonies.

09Bc11 is a field isolate collected in 2009 from strawberries. This isolate exhibits resistance to multiple fungicides, in particular iprodione resistance, conferred by the I365S mutation within the *bos1* gene, and boscalid resistance, conferred by the H272R mutation within *sdhB*. This isolate also displays the typical anilinopyrimidine (AP)-specific resistance phenotype Ani^R1^. We made use of the fully sequenced 09Bc11 x B05.10 mapping population and associated SNP dataset (Van Kan et al., 2017) to generate the Ani^R1^ mapping window. A total of 70 progeny isolates (35 resistant and 35 sensitive) were selected from a phenotyping test on agar plates, using a discriminatory CDL concentration of 10 mg·L^−1^. The resistance allele segregated in a Mendelian manner of 1:1 in the offspring, supporting a monogenic origin as previously reported by others for the Ani^R1^ phenotype (Hilber and Hilber-Bodmer, 1998). Conversely to the relatively narrow chromosome 1 mapping windows we have reported for boscalid and iprodione resistances (Van Kan et al., 2017), Ani^R1^ mapped within a large region of 133 kb (linkage of resistance ≥98%: 1,077,122–1,210,258) on chromosome 10 (Figure 2A), which encodes 31 genes in total. This region displays particularly low recombination rates as inferred from the relationship between physical and genetic distances (Van Kan et al., 2017). This may be caused by the presence of a predicted 35 kb centromeric region (1,132,000–1,167,000) at the center of the window.

An iterative reverse genetic procedure was used to further delimit the genetic factor conferring the Ani^R1^ phenotype (Figures 2B–D, see Materials and Methods, and Table S3). The smallest fragment, amplified from the resistant strain 09Bc11, that resulted in resistant colonies after transformation into the sensitive reference strain was Bcin10-1-2 (1,077,456–1,081,069; Figures 2C,D). The sole non-synonymous SNP within this region is located at position 1,078,999 on chromosome 10 and leads to an L412F substitution within the protein encoded by the gene Bcin10g02880 (termed *Bcpos5* hereafter). The encoded BcPos5 protein is the *Botrytis* ortholog of Pos5, the mitochondrial NADH kinase of yeast.

### Characterization of further Ani^R1^ field mutations

**Figure 3:**
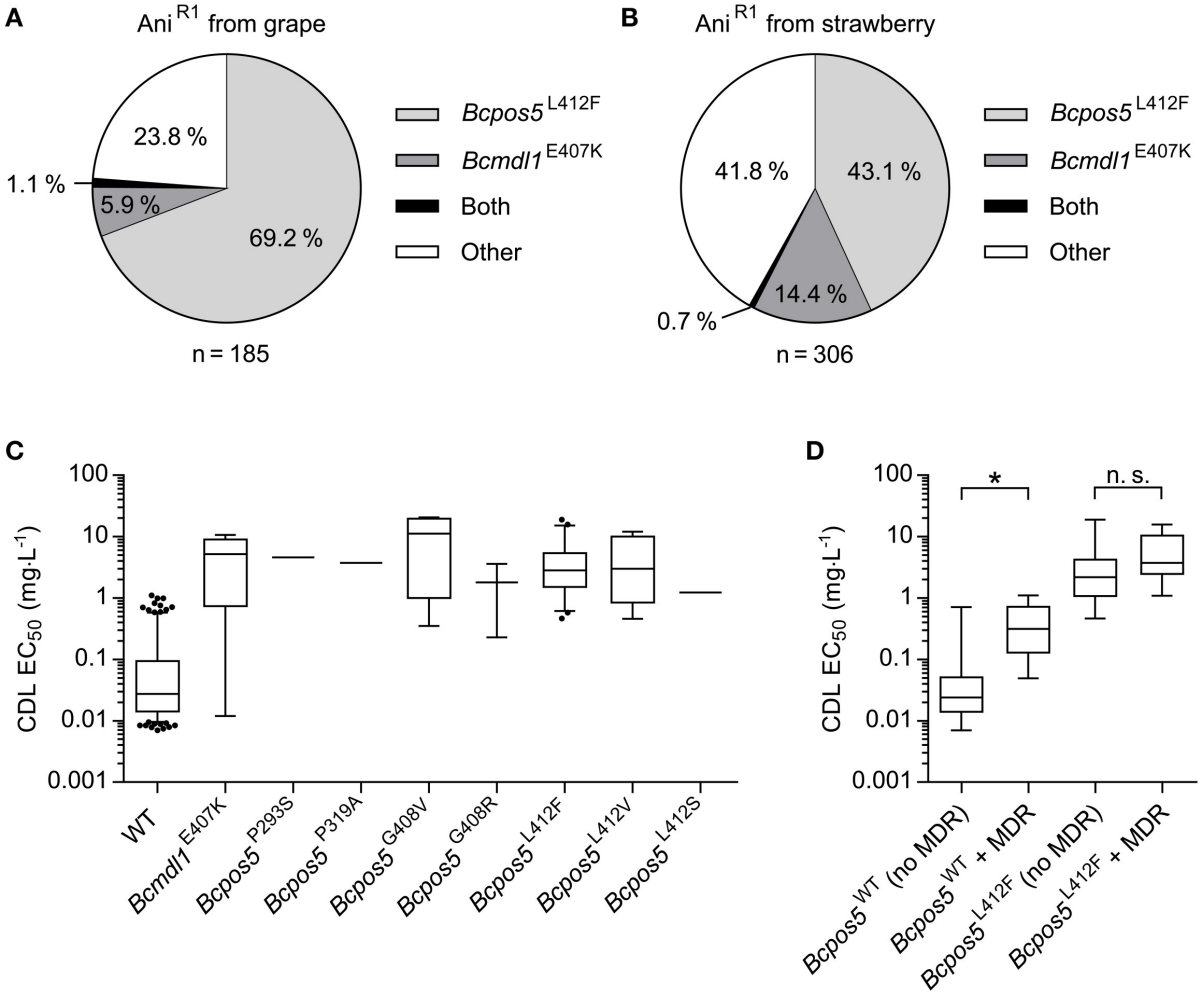
CDL resistance genotypes in *B. cinerea* monitoring populations. **(A,B)** Partition of *Bcpos5*^L412F^ and *Bcmdl1*^E407K^ as inferred from pyrosequencing within 491 Ani^R1^ samples collected between 2009 and 2014 from grape and strawberry, respectively. **(C)** Box-plot representation of the distribution of CDL sensitivity for the *Bcpos5* and *Bcmdl1* genotypes within the monitoring population isolated in 2014. The different genotypes were either detected by pyrosequencing (G408R/V, L412V/F, E407K, Table S1) or Sanger sequencing (Table S2). **(D)** Effect of MDR on the CDL sensitivity range for WT and *Bcpos5*^L412F^ genotypes based on the 2014 population (Table S1). Results of unpaired *t*-tests (GraphPad Prism 6.0) comparing *Bcpos5*^WT^ with or without MDR and *Bcpos5*^L412F^ with or without MDR (^*^p < 0.0001, n.s., not significant) are shown.

To characterize the frequency of the newly identified *Bcpos5*^L412F^ mutation in field populations an allele quantification test (Table S4) was run on Ani^R1^ field samples collected for the whole 2009–2014 period. This test enabled the detection of the L412F allele in 69.2 and 43.1% of Ani^R1^ samples originating from grape and strawberry, respectively (128 out of 185 from grape and 132 out of 306 from strawberry) (Figures 3A,B). A small number of samples displaying the Ani^R1^ phenotype but no L412F mutation nor signs of multidrug resistance (MDR) (EC_50_ values toward FDL <0.1 mg·L^−1^ were considered non-MDR), were subjected to Sanger sequencing of the set of resistance genes formerly identified by UV mutagenesis excluding *Bcmcr1* (Table S2). This resulted in the detection of the *Bcmdl1*^E407K^ mutation in Ani^R1^ field samples, a mutation which we initially identified in the UV mutant CDL10-34 (Table 1). *Bcmdl1*^E407K^ was found at a frequency of 5.9% in grape and 14.4% in strawberry (11 out of 185 samples from grape and 44 out of 306 samples from strawberry) (Figures 3A,B) as inferred from pyrosequencing allele quantification assay (Table S4). Samples carrying both alleles were also detected at a low frequency (about 1%). Together these 2 mutations could explain 76.2 and 58.2% of the Ani^R1^ phenotype from grape and strawberry samples, respectively (Figures 3A,B). However from this analysis, more than one third of the Ani^R1^ samples (35%) remained unexplained.

To further characterize the panel of possible Ani^R1^ mutations, we specifically focused on samples collected in 2014 and performed Sanger sequencing of the *Bcmdl1* and *Bcpos5* genes for a subset of unexplained resistant samples displaying otherwise pure genotypes according to the previously mentioned *Bcpos5*^L412F^ and *Bcmdl1*^E407K^ allele quantification assays. As a result, a range of novel BcPos5 variants were detected, mainly at the known L412 position, for which the new L412S and L412V variants were identified and at the nearby G408 position, for which the G408R and G408V mutations were identified. Novel genotyping assays were defined for these two positions (Table S4). The pyrosequencing approach was complemented with Sanger sequencing of *Bcmdl1* and *Bcpos5* for the last unexplained Ani^R1^ samples (Tables S2, S3). This iterative process enabled the characterization of the full 2014 population (Table S1). In addition to the above-mentioned G408R/V and L412F/V/S mutations three more amino acid substitutions within BcPos5: V273I, P293S, and P319A, and a novel amino acid substitution within BcMdl1: S466R were identified (Table 2).

**Table 2:** Validated and proposed CDL resistance-related mutations identified in field samples of *B. cinerea*.

***B. cinerea* gene ID**	**Trivial name**	**Codon**	**Amino acid change**	**Frequency within Ani^R1^ field isolates in 2014 (grape and strawberry)**
Bcin10g02880	*Bcpos5*	GTT▸ATT	V273I	Not found
		CCA▸TCA	P293S	<1%
		CCG▸GCG	P319A	<1%
		GGC▸CGC	G408R	3%
		GGC▸GTC	G408V	9%
		TTG▸TTC/T	L412F	68%
		TTG▸TCG^‡^	L412S	<1%
		TTG▸GTG	L412V	8%
Bcin16g00820	*Bcmdl1*	GAA▸AAA	E407K	10%
		AGC▸AGG	S466R	Not found

‡ *Not validated by reverse genetics. Frequencies of genotypes within field samples are based on the entire monitoring population of 2014, including samples displaying mixed WT-mutant genotypes (in such case counted as mutant)*.

Overall, the sensitivity values did not differ much across the different Ani^R1^ genotypes (Figure 3C). The median CDL sensitivity ranged between 1.22 mg·L^−1^ for *Bcpos5*^L412S^ and 11.23 mg·L^−1^ for *Bcpos5*^G408V^, which correspond to resistance factors of 45 and 416-fold, respectively. A very small proportion of the samples displayed no sensitivity shift in the bioassay (single test), despite one of the pure resistance alleles being detected by pyrosequencing (Figure 3C, sensitive outliers carrying *Bcmdl1*^E407K^). Conversely, samples displaying slightly CDL-shifted phenotypes (EC_50_ between 0.1 and 1 mg·L^−1^) in the bioassay did not contain any of the known *Bcpos5* or *Bcmdl1* mutations. Most of these samples were also shifted for FDL, suggesting the shift was at least partially caused by MDR (Figure 3D) (Kretschmer et al., 2009). Samples carrying both *Bcpos5*^L412F^ and MDR showed a slightly increased distribution range of EC_50_ values suggesting an additional effect of MDR on Ani^R1^ but this difference was not statistically significant (Figure 3D). Despite Ani^R1^ phenotypes could be explained for all samples collected from grape and strawberry in 2014, the characterization of confirmed Ani^R1^ samples from tomato suggests that additional resistance mechanisms outside *Bcmdl1, Bcpos5*, and MDR may occur in the field at a low frequency (Table S2).

**Figure 4:**
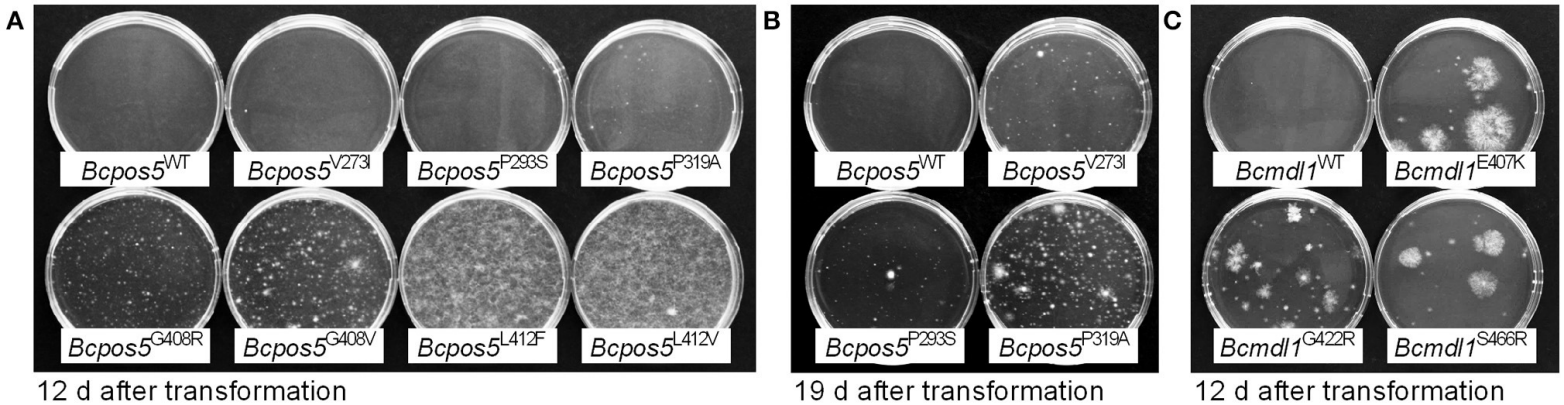
Validation of CDL resistance-conferring field and UV mutant genotypes in *Bcpos5* and *Bcmdl1* by reverse genetics. Result of a transformation of reference strain B05.10 protoplasts with 8 pmoles of PCR products amplified from genomic DNA of field isolates (UV mutant CDL50-8 in case of *Bcmdl1*^G422R^) carrying putatively resistance-conferring SNPs, on selective plates supplemented with CDL (10 mg·L^−1^). **(A,B)** *Bcpos5* PCR products transformed included the complete coding sequences. Pictures taken 12 or 19 days after transformation, respectively. **(C)** *Bcmdl1* PCR products, truncated at 5′ and 3′ ends.

All but one (L412S) of the *Bcpos5* mutations and the *Bcmdl1* field mutations E407K and S466R were validated by reverse genetics (Figure 4). The alleles displayed clear growth differences on selection plates after their transformation into CDL-sensitive protoplasts. In particular L412F and L412V transformants showed a much better protoplast recovery and faster growth on solid media, whereas V273I, P293S, and P319A colonies appeared after a longer period of incubation (Figures 4A,B). For reasons that are not understood the transformation of truncated versions of the *Bcpos5* gene followed by direct selection on CDL-amended plates failed, L412V being the only allele giving satisfactory results with this approach. In agreement with this, Sanger sequencing of individual transformants showed that the L412V mutation successfully replaced the wild type allele at the *Bcpos5* locus. Conversely, the wild type allele remained present for all other *Bcpos5* mutant alleles tested, suggesting that resistance was conferred by the ectopic integration of functional copies at unknown genomic loci in these transformants. In contrast, both E407K and S466R resistance-conferring mutations of *Bcmdl1* successfully replaced the wild type copy, indicating correct homologous recombination events in the transformants.

### Combination of the two major field resistance mechanisms does not increase resistance levels

**Figure 5:**
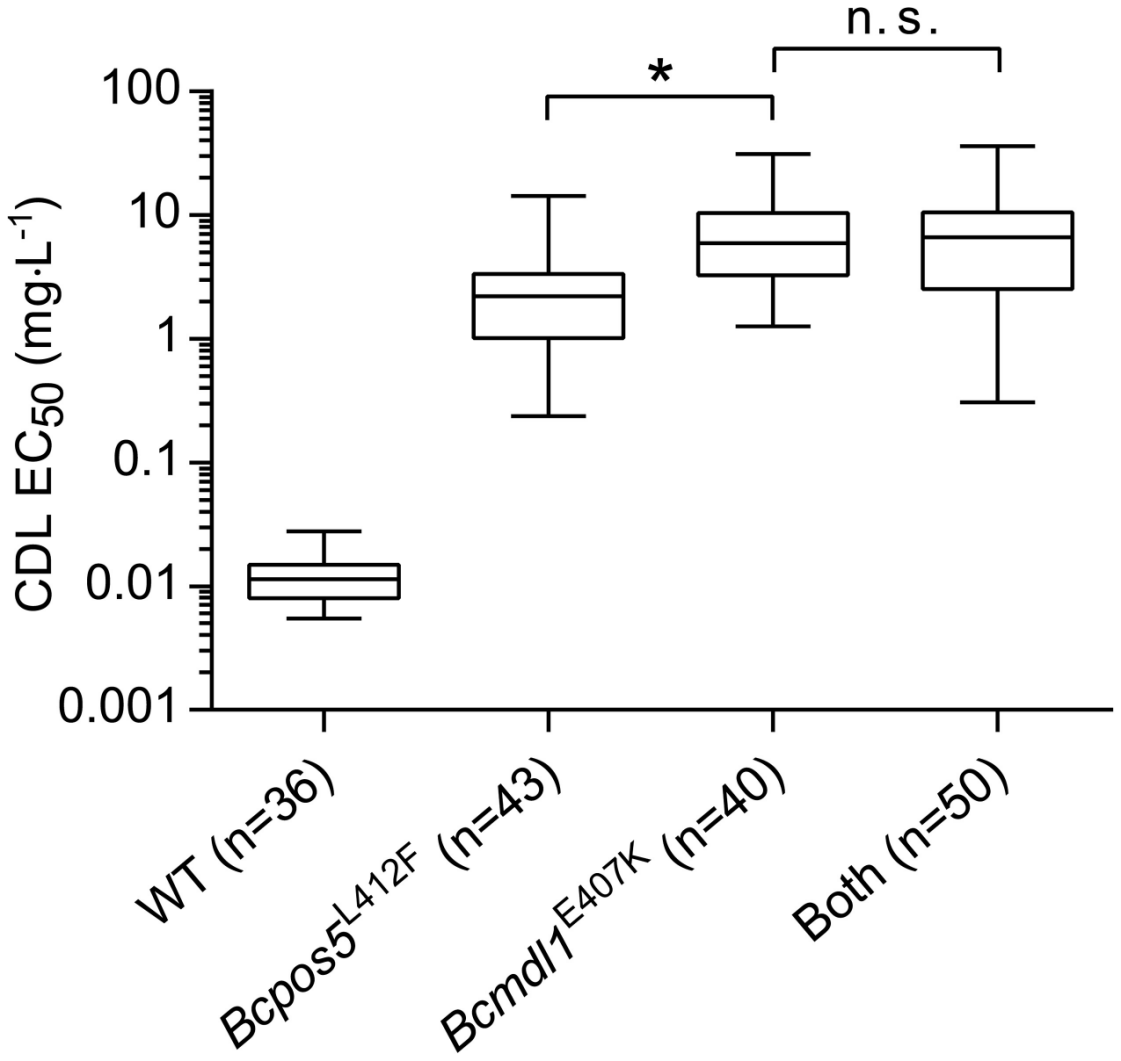
Phenotypes of progeny isolates from a cross between *Bcmdl1*^E407K^ and *Bcpos5*^L412F^. Box and whisker plots show EC_50_ values measured in technical duplicates (at BIOtransfer). Results of unpaired *t*-tests (GraphPad Prism 6.0) between two of the progeny genotypes are indicated (^*^p < 0.0001, n. s., not significant).

Some field samples displayed genotypes combining both *Bcmdl1*^E407K^ and *Bcpos5*^L412F^ mutations (Figures 3A,B). To understand whether or not these two resistance factors interact with each other and increase resistance levels beyond those observed for the single mutations, a mapping population of a cross between 09Bc11 (*Bcpos5*^L412F^) and BAR633 (*Bcmdl1*^E407K^) was produced and characterized genetically and phenotypically (see Materials and Methods). The resulting segregation pattern fit the Mendelian distribution expected for two independent alleles conferring resistance (WT: *Bcpos5*^L412F^: *Bcmdl1*^E407K^: Both = 36: 43: 40: 50). As shown in Figure 5, *Bcmdl1*^E407K^ conferred slightly but significantly higher resistance levels compared to *Bcpos5*^L412F^ (*p* < 0.0001), but the combined *Bcpos5*^L412F^ + *Bcmdl1*^E407K^ resistance levels were similar to those displayed by *Bcmdl1*^E407K^ alone (*p* = 0.99).

### The mutations in *Bcpos5* and *Bcmdl1* are cross-resistant toward different APs

**Figure 6:**
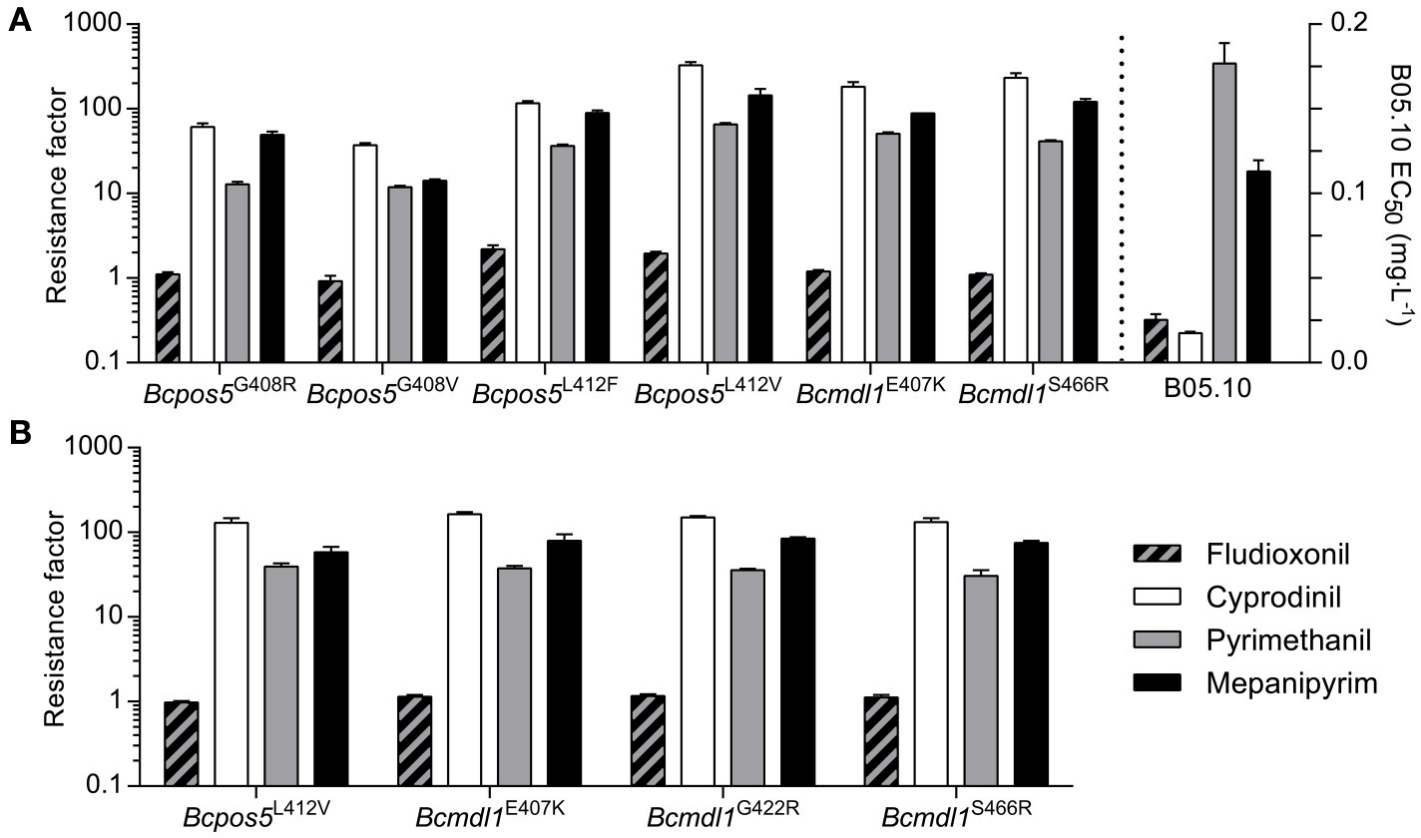
Cross-resistance profiles of a collection of isolates including the most prevalent AP resistance mutations from the field and available corresponding homologous recombinants. Resistance factors were calculated relative to the reference strain B05.10. Sensitivity to FDL was tested to confirm the absence of MDR1/3 (multidrug resistance) phenotypes. **(A)** Individual single spore isolates with *Bcpos5* and *Bcmdl1* genotypes confirmed by Sanger sequencing. *Bcpos5*^G408R^: 14-BC-183^s^, *Bcpos5*^G408V^: 11-BC-060^s^, *Bcpos5*^L412F^: 09Bc11, *Bcpos5*^L412V^: 14-BC-007^s^, *Bcmdl1*^E407K^: 14-BC-020^s^, *Bcmdl1*^S466R^: 10-BC-041^s^ (see Table S2). Mean values of technical triplicates ± standard deviation of one isolate per mutation are shown. **(B)** Homologous recombinants, mean values of three transformants per genotype ± standard deviation are shown. Individual transformants were tested in technical triplicates.

Target site mutations frequently confer slightly different cross-resistance patterns toward different molecules within the same class of mode of action. Differences in cross-resistance patterns across the active substances are caused by slightly different binding modes and contrasted binding perturbations resulting from mutations within the inhibitor binding pocket. For example, within the succinate dehydrogenase inhibitor (SDHI) fungicides, different cross-resistance patterns, and even negative cross-resistance has been observed across chemicals and mutation pairs (Scalliet et al., 2012; Laleve et al., 2014). AP fungicides are very tightly structurally related to one another but we questioned whether or not frequent *Bcpos5* or *Bcmdl1* mutations may display some hint of differential resistance. As shown in Figure 6A, all tested field isolate genotypes conferred clear cross-resistance toward the three tested APs, with CDL showing both the strongest intrinsic activity on the wild type and the highest resistance factors in the mutants. According to the tested field isolates, the strain carrying *Bcpos5*^G408V^ was leading to the weakest resistance (resistance factor of 37-fold for CDL) which is contrasting to the median sensitivity derived resistance factor in field isolates (resistance factor of 416-fold, Figure 3) but the high frequency of MDR in these isolates was noteworthy and may explain this difference (Table S1). Based on the tested field isolates there was no obvious sign of a strong differential cross-resistance pattern across the three APs and mutation types (Figure 6A). Cross-resistance was also observed in the available homologous recombinants (Figure 6B). Resistance factors as well as cross resistance patterns were very similar for the three tested genotypes *Bcmdl1*^E407K^, *Bcmdl1*^G422R^, and *Bcmdl1*^S466R^ (Figure 6B). Despite not displaying signs of MDR, the tested *Bcpos5*^L412V^ field isolate showed almost 3-fold higher resistance factors compared to its cognate B05.10 homologous recombinants (Figures 6A,B) evidencing the important role of individual genetic backgrounds in AP sensitivity. However, it can be noticed that the sensitivity ratios across APs were maintained between the *Bcpos5*^L412V^ field isolate and its cognate homologous recombinants. *Bcpos5* field mutants displayed minor differences in sensitivity ratios across APs (compare resistance factors for mepanipyrim and pyrimethanil in *Bcpos5*^G408V^ and *Bcpos5*^G408R^, Figure 6A), but given the importance of genetic background additional homologous recombinants for *Bcpos5*^G408R/V^ and *Bspos5*^L412F^ will be required to validate these potential variations in cross resistance profiles.

## Discussion

### Outcome of UV mutagenesis, stability of resistance, and dose response phenotypes

We report the discovery and validation of anilinopyrimidine (AP) resistance-conferring mutations within nine distinct nuclear genes, among which eight were identified from our laboratory mutant collection. Only one of these genes, *Bcmdl1*, was also found to be responsible for a proportion of the Ani^R1^ resistant phenotypes in field isolates. This result suggests that the majority of the mutations found by UV mutagenesis screening may negatively affect the fitness of *B. cinerea* and prevent the successful propagation of the corresponding resistance alleles in field populations. Practical observations inferred from our UV mutant selection support the occurrence of additional factors, because a high proportion of the selected isolates, able to grow on primary and secondary selection plates, did not show clearly shifted profiles in liquid culture assays afterwards. In some cases resistance was even lost during the second round of selection. This genetic and phenotypical instability has been reported by others for *in vitro* generated AP-resistant mutants of *Botrytis* (Leroux et al., 2002), suggesting that in these primary mutants resistance may be caused by mutations affecting essential genes and encompassing a very high fitness cost, even under *in vitro* conditions. A shift back to sensitivity may occur if the mutated nuclei (or mitochondria) were not transmitted to the spores used for the inoculation of secondary selection plates. *B*. *cinerea* is a multinucleate pathogen, therefore the loss of function of essential genes is usually partially tolerated as long as some of the nuclei still carry the wild type allele to compensate (Giesbert et al., 2012). This might also explain why, in our CDL-resistant isolate CDL10-11, only a proportion of the sequencing reads showed non-synonymous mutations within multiple genes. One of the candidate mutations, resulting in a premature stop codon, was found in the ortholog of the gene encoding the yeast mitochondrial inner membrane ADP/ATP carrier Pet9. Considering the mitochondrial localization of the validated resistance-related genes, this likely loss of function mutation may be responsible for CDL resistance in this isolate, possibly through a gene dosage effect, but a reverse genetic experiment failed to confirm this hypothesis.

Our characterized laboratory mutants displayed striking phenotypical variation of fungicide dose-response curves (Figure 1 and Figure S2), but all resistant strains were still at least partially controlled at concentrations of CDL above 10 mg·L^−1^. This maintained activity may be related to secondary mode(s) of action at high doses of the active ingredient or to a partial inhibition at the molecular target site in the mutants. In our assay conditions CDL EC_50_ values varied between 2.8 and 5.4 mg·L^−1^ (Table 1) for all UV mutants tested, which contrasts to some field isolates displaying EC_50_ values above 20 mg·L^−1^ (Table S1). We suspect that additional factors play a role in field isolates, in particular MDR combined with Ani^R1^ shifts the sensitivity to higher values (Figure 3D).

Several UV strains, and in particular the *Bcatm1* mutants (Figure 1C and Figure S2), displayed clear biphasic dose-response curves, even in mutants shown to be pure from the sequence data (Table S6). Very minor signs of biphasic dose-responses were observed for *Bcafg3, Bcmdl1* (mostly for the G422R mutation), *Bcmix17, BcoliC*, and *Bcphb2*. Finally, a so far unique “displaced” biphasic dose-response (Figure 1D) was displayed by two different frameshift mutants of *Bcmcr1*, which both affect the C-terminal part of the protein and lead to polypeptide sequences that are 15 amino acids longer (Figure S1). These biphasic behaviors suggest a complex interplay between the primary mode of action and the likely suppressive/compensatory mechanisms caused by the identified mutations in these genes.

We assume that dose-response curves of the mutants may be indicative of the functional distance between the mutated gene product and the primary target. We would expect from target binding site mutants to directly suppress growth inhibition at low doses of the fungicide, resulting in monophasic dose-responses. On the contrary, biphasic dose-responses show that a primary inhibitory effect at low fungicide concentrations is not prevented. This is suggesting that in such cases resistance is conferred by a suppressive effect or compensatory mechanism functionally related to the molecular target. If this is true, candidates for the primary target would include *Bcpos5* and *Bcdnm1*, both of which displayed no sign of biphasicity in our assays. Among these, *Bcpos5* was found to explain most of the Ani^R1^ resistance in the field populations, but was not identified in our UV mutant collection. In theory, resistance through mutated versions of this gene should have been found in our UV screening. Interestingly, a wide range of *Bcpos5* mutations were shown to confer resistance in reverse genetic experiments, but homologous recombination was only observed for one allele (L412V), whereas ectopic integrations were obtained for all other alleles tested. These results might indicate that the resistance phenotype caused by the majority of *Bcpos5* mutations would have required more time to be fully expressed than was allowed by the direct selection of UV mutants and transformants on agar plates supplemented with CDL. However, this hypothesis will require further validation. It is also possible that mutants in *Bcpos5* occurred at a low frequency in our UV screening, but were not detected, because not all isolates were genetically characterized.

**Figure 7:**
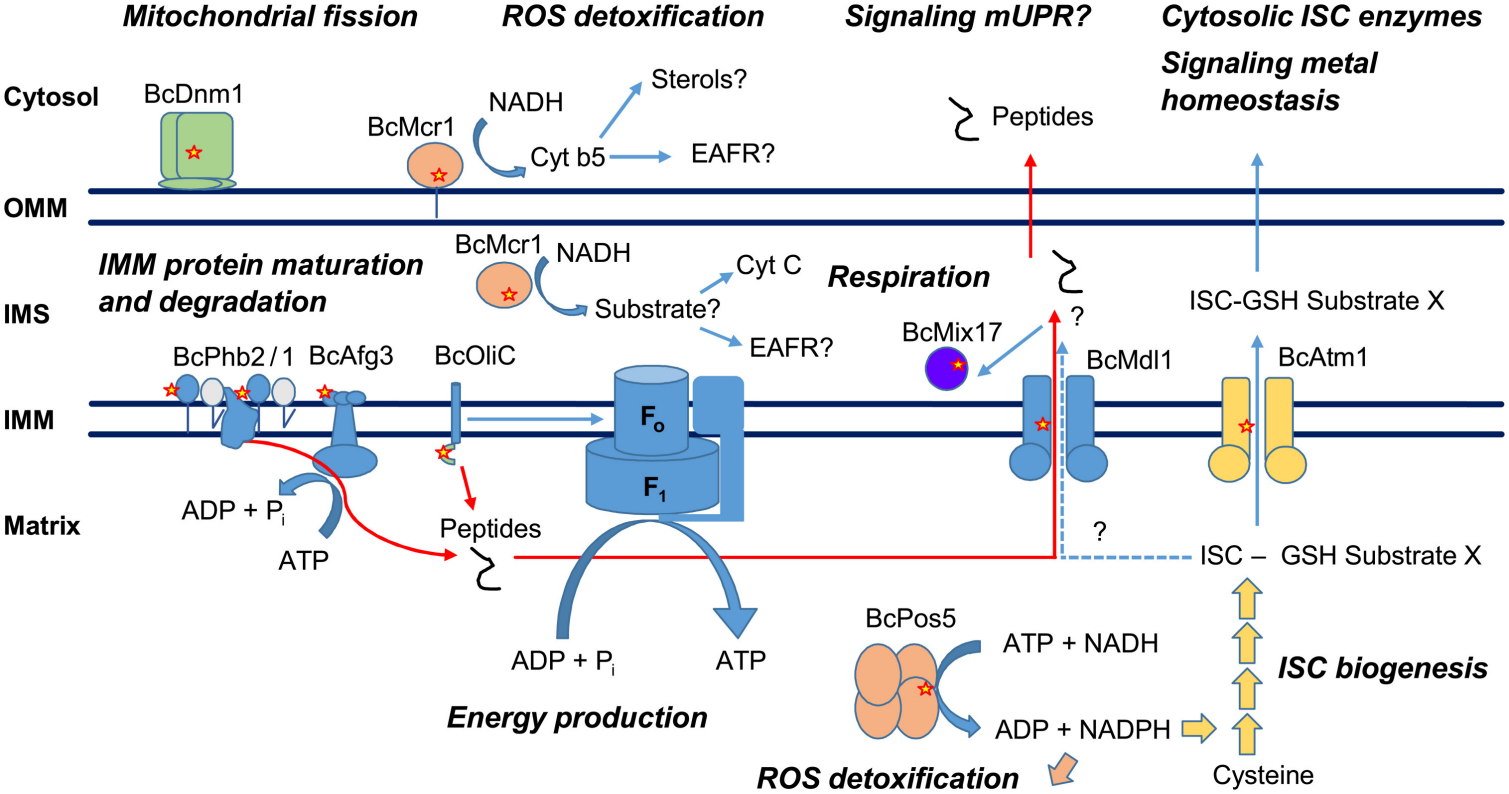
Model of hypothetical interactions between the CDL resistance-related mitochondrial proteins. OMM, Outer mitochondrial membrane; IMS, Intermembrane space; IMM, Inner mitochondrial membrane; ISC, Iron sulfur cluster; mUPR, Mitochondrial unfolded protein response; EAFR, D-erythroascorbyl free radicals. Positions of resistance-related mutations are marked with red stars.

All 9 CDL resistance-related genes identified in this study are nuclearly-encoding proteins targeted to the mitochondria and involved in various mitochondrial functions (Figure 7, Table 3), suggesting that the mitochondrion is the primary target of this class of fungicides. Interestingly, some of these proteins have been functionally connected and shown to either physically or genetically interact in yeast and other systems. This is particularly striking for Phb2, Afg3, Mdl1, and Atp9 (BcOliC), the oligomycin resistance-conferring subunit c of the F_1_F_O_ ATP synthase. Phb2, Afg3, and Mdl1 are involved in mitochondrial respiration and energy production through their role in the folding, maturation and degradation of internal mitochondrial membrane (IMM) proteins, and in the transport of peptidic degradation products by Mdl1 from the mitochondrial matrix to the intermembrane space (IMS).

**Table 3:** Homologous gene products of CDL resistance-related *B. cinerea* genes and their reported cellular functions.

***B. cinerea* gene accession no**.	**Yeast homolog**	**Cellular function in yeast**
Bcin01g01830	Mix17	Mitochondrial intermembrane space protein carrying a twin Cx_9_C motif and a DUF2076 domain, exact function unknown, knock-out shows significant respiratory deficiency
Bcin02g02630	Dnm1	Dynamin-related GTPase involved in mitochondrial fission and morphology, participates in endocytosis and regulation of peroxisome abundance
Bcin03g02170	Afg3	Mitochondrial inner membrane AAA protease, mediates degradation of misfolded or unassembled proteins, essential for the biogenesis of respiratory chain complexes
Bcin07g01710	Phb2	Prohibitin2 forms ring-like supercomplexes with Phb1, regulates degradation of mitochondrial inner membrane proteins by m-AAA proteases (like Afg3)
Bcin10g00060	Mcr1	Mitochondrial NADH-cytochrome b5 reductase, proposed role in sterol biosynthesis pathway, reduction of D-erythroascorbyl free radicals link to oxidative stress tolerance
Bcin10g01500	OliC	Subunit c of the F_O_ (oligomycin sensitive) part of the mitochondrial F_1_F_O_ ATP synthase
Bcin10g02880	Pos5	NADH kinase, production of mitochondrial NADPH from NADH and ATP, required for efficient ISC biogenesis and ROS defense by regeneration of major antioxidants
Bcin15g00830	Atm1	Mitochondrial inner membrane ABC transporter, involved in mitochondrial iron homeostasis and biogenesis of cytosolic iron sulfur cluster proteins
Bcin16g00820	Mdl1	Mitochondrial inner membrane ABC transporter, putatively involved in export of peptides derived from degradation of proteins, physical interaction with F_1_F_O_ ATP synthase

*References for all yeast homologs are listed in the text*.

### Possible functional connections between AP resistance-conferring genes

#### Prohibitin 2 and the m-AAA protease Afg3

The yeast prohibitins Phb2 and Phb1 (likely the product of Bcin14g04870 in *Botrytis*) are proteins of the internal membrane of the mitochondria, forming ring-like supercomplexes (Tatsuta et al., 2005). These complexes associate with nascent polypeptides and regulate their degradation, acting like chaperones (Nijtmans et al., 2000). The deletion of prohibitins leads to accelerated degradation of non-assembled inner membrane proteins by the m-AAA proteases to which Afg3 belongs (Steglich et al., 1999), indicating a negative regulatory effect of prohibitins on the m-AAA protease activity. The BcPhb2^L153S^ CDL resistance mutation is predicted to occur within a helix associated with protein-protein interactions and orientated toward the IM space. Therefore, this mutation may either impact the formation of the prohibitin supercomplex or its interaction with other proteins, such as the m-AAA protease Afg3 or misfolded proteins, and influence their degradation. In yeast, striking genetic interactions have been observed between prohibitins and the regulation of Atp23, a conserved IMS metallopeptidase involved in the maturation of Atp6 and to its association with Atp9, the subunit c of the F_1_F_O_ ATP synthase (Osman et al., 2007). The functionality of the pea m-AAA protease PsFtsH, heterologously expressed in yeast, is necessary for the accumulation of Atp9 within mitochondrial membranes, suggesting a more direct involvement in the maturation of Atp9 (Kolodziejczak et al., 2002). These interactions establish a connection between the prohibitins, the m-AAA protease Afg3 and Atp9. Afg3 is one of the two m-AAA proteases in yeast (Yta12 being the other). These enzymes are located within the internal mitochondrial membrane and mediate the degradation of misfolded or unassembled proteins. In yeast, Afg3 is associated with Yta12 and acts as a heterohexamer. The enzyme is integrated within the IMM and its ATP-dependent proteolytic site is orientated toward the matrix (Paul and Tzagoloff, 1995; Lee et al., 2011). In the B05.10 genome no other m-AAA protease is predicted, therefore the complex is likely to be a homohexamer as found in *Neurospora crassa* (Klanner et al., 2001). The proteolytic function of the m-AAA proteases has been shown to be essential for the biogenesis of respiratory chain complexes, because inactive forms lead to respiration deficiency (Arlt et al., 1996, 1998). This phenomenon is thought to be mostly caused by deficient intron splicing of mitochondrial RNA transcripts by the mRNA maturases, a process which is under proteolytic control by the m-AAA proteases (Arlt et al., 1998). In addition, Afg3 loss of function mutants fail to properly assemble the F_1_ subunit of the F_1_F_O_ ATP synthase (Paul and Tzagoloff, 1995). The BcAfg3^L305P^ mutation, conferring AP resistance, corresponds to L178 and L236 of Yta10 (Afg3) and Yta12 of yeast, respectively. The position is predicted to be located within a domain exposed to the intermembrane space and likely to interact with prohibitins or its substrates (Ramelot et al., 2013). The mutation may therefore functionally mimic BcPhb2 mutations if the BcPhb2-BcAfg3 interaction effectively accounts for resistance.

#### The mitochondrial ABC transporter Mdl1 and its interaction with the F_1_F_O_ ATP synthase

Mdl1 is a “half type” ABC transporter, the active form of the enzyme being a homodimer (Hofacker et al., 2007). Mdl1 is involved in the transport of peptides of a molecular mass between 600 to 2,100 Da (6 to 20 amino acids), derived from the degradation of mitochondrial proteins (Young et al., 2001). Mdl1 was shown to reversibly associate to the functional F_1_F_O_ ATP synthase *in vivo* (Galluhn and Langer, 2004), depending on the ATP concentration and on the F_1_F_O_ ATP synthase activity. The physical interaction between Mdl1 and ATP synthase involves membrane imbedded parts of Mdl1 and the F_O_ sector of the ATP synthase, which contains the oligomycin-sensitivity conferring subunit c (Atp9) in which we observed the CDL resistance-related mutation. Interestingly, Galluhn and Langer (2004) reported a reversed ATP dependence in the presence of oligomycin, which they suggested to be due to a different conformational state of the ATP synthase, affecting the interaction. This suggests a direct interaction between Mdl1 and the oligomycin-sensitivity conferring subunit c. It has been proposed that the association of Mdl1 with the ATP synthase regulates Mdl1-mediated peptide export, the unbound homodimer being the active form (Galluhn and Langer, 2004).

Aside from the above-mentioned Mdl1-ATP synthase interaction, one of the AP-resistant UV isolates carried an R33C mutation within the N-terminal mitochondrial targeting presequence of the nuclearly encoded BcOliC. This targeting peptide is predicted to be cut off from the polypeptide after amino acid 68 based on sequence similarity to *N*. *crassa* and *Aspergillus nidulans* (Ward and Turner, 1986); Y69 and the immediate downstream sequence of BcOliC align well with the first tyrosine and subsequent amino acids of the mature subunit c proteins in these species. The functional impact of this unique R33C mutation within the 68 amino acids of the presequence of BcOliC is not clear. This mutation leads to the exchange of one charged basic residue to a neutral cysteine and may possibly influence cytosolic precursor protein recognition by the TOM complex. Reduced import of subunit c could affect overall ATP synthase assembly. Interestingly, conversely to yeast in which Atp9 is solely encoded in the mitochondrial genome, *B*. *cinerea* carries two genes encoding this subunit, one mitochondrial and one nuclear. In *Podospora anserina* there is no mitochondrial copy but subunit c is encoded by two nuclear genes that can be swapped but are tightly developmentally regulated throughout the life cycle of the fungus (Dequard-Chablat et al., 2011). In mammals the ATP synthase subunit c has three isoforms differing solely in their cleavable targeting peptides. These peptides, in addition to mediating mitochondrial protein import, were shown to have a major role in respiratory chain maintenance (Vives-Bauza et al., 2010). It is tempting to speculate that the R33C mutation of the BcOliC targeting peptide leads to AP resistance by interfering with an additional biological function carried by the targeting peptide itself. This will, however, require further investigation.

#### Mdl1, signaling, and potential links to the m-AAA protease Afg3

Other features connect Mdl1 to Afg3. First, radiolabeling studies suggest that Mdl1 supports the transport of Afg3 m-AAA protease-derived peptides, because the export of long peptides is greatly diminished in Δ*afg3* mutants (Young et al., 2001). Second, m-AAA protease activity is essential for the formation of a functional F_1_F_O_ ATP synthase (Paul and Tzagoloff, 1995; Arlt et al., 1998), linking Afg3 to the above mentioned Mdl1-F_1_F_O_ ATP synthase interaction. Afg3 and Mdl1 are therefore strongly functionally connected and could be regulating each other in coordination with ATP synthase activity. A 2.3 nm resolution structure of yeast Mdl1 was inferred from single particle electron microscopy and refined by homology modeling, enabling us to determine the positions of the *B*. *cinerea* BcMdl1 resistance-conferring mutations within the yeast tridimensional model (Hofacker et al., 2007). BcMdl1 mutations E407K, G422R, and S466R correspond to positions E332, G348 and S391 in yeast. All are located within transmembrane domain helixes (TMH) of the transporter, E332 and G348 are within TMH5 and S391 within TMH6. These amino acids are located at positions predicted to face the interior of the channel and may form part of the substrate binding cavity (Schaedler et al., 2015). Therefore, CDL resistance-conferring mutations of BcMdl1 may have an influence on transported substrate preference or the kinetics of transport, which may in turn have a strong biological relevance. Indeed, peptide substrates exported by Mdl1 trigger mitochondrial to nuclear signaling, as suggested from gene expression profiling of Δ*mdl1* and Δ*yme1*Δ*mdl1* yeast mutants (Arnold et al., 2006). In *Caenorhabditis elegans*, genetic analysis of genes suppressing the mitochondrial unfolded protein response signaling pathway (UPR^mt^) identified Haf1, an ortholog of Mdl1 (Haynes et al., 2010). Haf1 was shown to be essential for channeling peptides derived from degraded mitochondrial proteins from the mitochondria to the cytosol and leading to the re-localization of a bZIP protein (ZC376.7) from the cytosol to the nucleus. Although such mitochondrial to nuclear peptide signaling has never been demonstrated in *B*. *cinerea* one can hypothesize a role of the resistance-conferring mutations of BcMdl1 in interfering with the transport of particular peptides or signaling molecules. One can also hypothesize that the BcMdl1 transporter specificity is modified in a way that effectively supports the transport of AP molecules out of the mitochondrial matrix in resistant BcMdl1 mutants. To our knowledge, this would be the first example of mitochondrial multidrug resistance.

#### The mitochondrial ABC transporter Atm1 and its connection to iron sulfur cluster biogenesis

Mdl1 also shows a clear genetic interaction and functional connectivity with another CDL resistance-conferring gene, Atm1. In yeast, Atm1 null mutants accumulate mitochondrial iron and show oversensitivity to H_2_O_2_. This phenotype is reverted by the overexpression of Mdl1, suggesting that Mdl1 could support, although less effectively, the transport of Atm1 substrates (Chloupkova et al., 2003). Atm1 is another half type ABC transporter similar to Mdl1 and is essential for mitochondrial iron homeostasis and for the biogenesis of cytosolic iron sulfur cluster (ISC) proteins (Kispal et al., 1997, 1999). Cell biology data and functional characterization demonstrate that Atm1 exports a sulfur-containing molecule generated by the mitochondrial ISC assembly machinery (Lill, 2009). In particular, transportomics studies with heterologously expressed protein suggest glutathione or glutathione polysulfite derivatives as substrates (Schaedler et al., 2014). The crystal structure of Atm1 was recently determined with reduced glutathione (GSH) bound (Srinivasan et al., 2014). The CDL resistance-conferring BcAtm1^E414K^ mutation corresponds to position D398 of Atm1 in yeast and E433 of Abc7 in human. Yeast D398 is located in the TMH6 of the protein and in direct interaction with the GSH C-terminus in the structure. This position is of particular interest because the E433K mutation of the human Abc7 is responsible for XLSA/A cerebellar ataxia (Bekri et al., 2000). The mutation leads to reduced activity in the maturation of cytosolic ISC proteins both in yeast and human, which strongly suggests BcAtm1^E414K^ should lead to a less active ISC export in *B*. *cinerea*. The exact substrate for Atm1 has not yet been identified but a glutathione derivative with coordinated ISC (ISC-GSH-SubstrateX in Figure 7) has been proposed (Srinivasan et al., 2014).

#### The mitochondrial NADH kinase Pos5 and its roles

Mitochondrial iron sulfur biosynthesis clearly links *Bcatm1* and *Bcpos5*, a gene responsible for the great majority of Ani^R1^ phenotypes in the field. Yeast Pos5 is a mitochondrial NADH kinase, an enzyme responsible for the production of the mitochondrial pool of NADPH from mitochondrial NADH and ATP (Outten and Culotta, 2003; Bieganowski et al., 2006; Miyagi et al., 2009). Pos5 was identified in screens for genes whose loss of function led to oversensitivity to ROS caused by H_2_O_2_, paraquat or hyperoxia (pos), and increased the mutation rate of mitochondrial DNA (Krems et al., 1995; Outten and Culotta, 2003; Strand et al., 2003). These phenotypes can be explained by the key role of mitochondrial NADPH in the regeneration of major antioxidants, i.e., reduced thioredoxin and glutathione, by NADPH-dependent reductases (Toledano et al., 2013). In addition, Pos5 is also required for efficient ISC biogenesis, and its enzyme activity appears to be a limiting factor in the pathway. The role of Pos5 in ISC biogenesis appears to be distinct from its function in anti-oxidant defense (Outten and Culotta, 2003; Pain et al., 2010). This not only links Pos5 to the regeneration of the reduced glutathione moiety of the Atm1 substrate, but to the formation of its ISC derivative. The structure of Pos5 complexed with NAD^+^ has been determined at a 2 Å resolution, enabling us to physically position CDL resistance mutations within the model (Ando et al., 2011). The enzyme is a soluble homotetramer, and the most frequent Ani^R1^ genotypes, mutated at positions F412 and G408 in BcPos5, correspond to positions L397 and G393 of yeast Pos5, respectively. These positions are located within a short distance (8 and 9 Å, respectively) from the NAD^+^ ribose phosphorylation site, suggesting that these mutations could have an influence on catalysis. Interestingly, the Pos5 ATP binding site has not yet been described and these residues may also be involved in the binding interaction with ATP. Other, less frequent field mutations, such as V273I, P293S, and P319A of BcPos5 correspond to positions I243, P263, and P289 in yeast Pos5, respectively. They are located at more distant sites and could possibly interfere with the formation of the tetramer or its potential allosteric regulation. Given the degree of connectivity of Pos5 with multiple other biochemical pathways, and the enzymatic substrates or products of many of the resistance genes we identified, it is tempting to hypothesize that this enzyme is the primary target for AP fungicides. Further work focusing on this aspect is ongoing.

#### Other AP resistance-related gene products: Mix17, Mcr1, and Dnm1

Finally, additional AP resistance-conferring mutations identified in our UV screening were located within *Bcmix17, Bcdnm1*, and *Bcmcr1* genes. However, the function of these genes appears less clearly connected to the core pathways described above.

BcMix17 is similar to Mix17 from yeast, with a slightly lower predicted molecular weight of 16.3 kDa compared to 17 kDa, in yeast. Mix17 is an intermembrane space (IMS) protein carrying a twin Cx_9_C motif, which acts as a signal for IMS targeting and is a substrate of the redox-driven MIA import pathway (Gabriel et al., 2007; Longen et al., 2009). The exact function of Mix17 has not yet been elucidated. The knock out in yeast is not lethal, grows normally on non-fermentable media, but shows significant respiratory deficiency (Longen et al., 2009). Interestingly, the G79D and G83E mutations occurred within a domain (DUF2076) with homology to periplasmic ligand-binding sensor proteins in bacteria (Marchler-Bauer et al., 2017). We hypothesize a possible link between these mutations and the sensing of substrates transported by BcMdl1 or BcAtm1.

BcMcr1 is similar to Mcr1, the unique mitochondrial NADH-cytochrome b5 reductase in yeast. Although the *mcr1* gene encodes one single protein targeted to the mitochondria, it is differentially processed into two isoforms localizing within two different sub-mitochondrial compartments (Hahne et al., 1994). The long isoform is anchored on the outer face of the outer mitochondrial membrane (OMM) through its amino terminal anchor sequence (Meineke et al., 2008). The IMS isoform uses the translocase of the outer membrane (TOM) complex to reach the inner membrane where it is processed to a shorter soluble IMS protein. The exact functions of the NADH-cytochrome b5 Mcr1 protein, and in particular the exact biological reason for this dual targeting, are not yet fully understood. Enzymatic studies suggested a role in supporting particular reducing steps within the sterol biosynthesis pathway (Lamb et al., 1999). The gene was shown to play a crucial role in the reduction of D-erythroascorbyl free radicals in *Saccharomyces cerevisiae*, pointing at a major role of the protein in mediating resistance toward oxidative stresses (Lee et al., 2001). It has been proposed that the IMS form may transfer electrons from external NADH to cytochrome c and therefore support antimycin-insensitive energy coupled oxidation of NADH (Hahne et al., 1994). Our AP-resistant BcMcr1 mutants were frameshift variants of the C-terminal part of the protein, which was extended by 15 residues for both mutants identified. These frameshifts are unlikely to influence the dual sub-organellar localization of the protein, which is triggered by its N-terminal part, but might impact enzyme kinetics or the nature of the reduced substrates, which could be different depending on the sub-organellar localization. The very unique growth inhibition dose-response curves displayed by these variants (Figure S1) suggest a complex interplay between the mode of action of APs and the concentration of substrates or products of this enzyme.

The last gene with less clear connections encodes BcDnm1, an ortholog of the dynamin-related GTPase Dnm1 of yeast, which regulates mitochondrial fission and also participates in endocytosis and regulates peroxisome abundance (Kuravi et al., 2006). Mitochondrial dynamics in the Δ*dnm1* yeast mutant are strongly affected, such that the mitochondrial network is converted into a planar net of interconnected tubules (Bleazard et al., 1999). The BcDnm1^E450G^ resistance-conferring mutation is located within the M domain of the protein, which is important for intramolecular interaction within the Dnm1 monomer of the functional protein homodimer. The vicinity of the *B*. *cinerea* mutation to the human Python mutation suggests it could similarly lead to a partial loss of function and consequently affect mitochondrial enzymes and ATP levels (Ashrafian et al., 2010), linking the Dnm1 resistance mutation to energy production. However, Dnm1 loss of function mutants of *P. anserina* and yeast displayed increased life span and showed no clear effects on neither mitochondrial membrane potential nor fitness (Scheckhuber et al., 2007). It will be interesting to observe whether AP treatment affects the mitochondrial morphology of *B*. *cinerea*, because a BcDnm1 loss of function mutation may counteract potential mitochondrial fragmentation and/or mitophagy triggered by the treatment (Nieto-Jacobo et al., 2012).

### General conclusions

Altogether, the identification of 9 different mitochondrial proteins conferring resistance to AP fungicides very strongly supports the hypothesis of a mitochondrial target. This is an unexpected finding given the lack of report of any effect on mitochondrial respiratory function of this fungicide class. Also, there is no direct connection between these genes and methionine biosynthesis, which has been considered to be the targeted pathway. NADPH, which is required for two steps of the methionine biosynthesis pathway, may connect to Pos5, but these steps are carried out within the cytosol. The required NADPH molecules are derived from the combined activity of the cytosolic NAD kinases (Bieganowski et al., 2006) and of the pentose phosphate pathway (for review see Thomas and Surdin-Kerjan, 1997). This suggests that the methionine reversal observed in *B*. *cinerea* may be the result of an antagonistic effect caused by the intracellular concentration of methionine. Furthermore, methionine acts as a regulatory hub in yeast, regulating multiple processes, such as cell division, oxidative stress response, and survival under starvation (Petti et al., 2011, 2012). In particular, a connection between the methionine-regulated transcription factors Met31 and Met32 and the regulation of iron-homeostasis genes involved in ISC biogenesis was demonstrated, which would link methionine reversal to this particular branch of the chemical-genetic interaction (Petti et al., 2012).

We report resistance toward one single class of fungicides caused by mutations within at least 9 different genes, suggesting novel functional connections that are likely to be conserved across species and therefore extremely relevant for cell biology. Such a diversity of possible resistance mechanisms for a single fungicide class is, to our knowledge, unique. From this study, and despite the fact that the majority of Ani^R1^ field resistances could be explained, it is clear that we did not identify all possible resistance mechanisms. It is likely that other mutations within other genes, yet to be discovered, can counteract AP fungicide activity in *B*. *cinerea*. Within our current set of genes, *Bcpos5*, encoding the mitochondrial NADH kinase, would constitute a good candidate for being the primary molecular target of AP fungicides. However, the very similar cross-resistance profiles across a panel of *Bcpos5* mutants did not suggest strong differential binding interaction across the different fungicides and mutant pairs, which is in accordance with the very similar structure carried by APs. Therefore, further work will be necessary to confirm the molecular target and the exact mechanism of action of this important class of fungicides. In particular, a better knowledge of the relationships between the molecular target and the multiple resistance mechanisms will be required to understand whether or not the various resistance mutations represent a gain or a loss of function within the identified resistance genes.

From a practical perspective, the identification of AP resistance-conferring mechanisms within field populations of *B*. *cinerea* will facilitate the development of quantitative molecular assays for resistance monitoring. This new knowledge will also enable the assessment of the stability and fitness of the different resistant variants of *Bcpos5* and *Bcmdl1*. Since resistance toward AP fungicides has also been described in other species and is in many cases infrequent within populations, it will be interesting to determine whether similar genes are affected or not and whether such resistance mechanisms encompass species-specific fitness costs.

## Author contributions

GS and AM conceived the project and designed the experiments. AM, DE, and GS conducted the experiments. AF, RD, and SW designed and performed next generation sequencings and did the bioinformatic analysis. TB designed and supervised the *Botrytis* monitoring (phenotyping assays) at BIOtransfer. AM, GS, RD, AF, SW, and AC wrote and reviewed the manuscript.

### Conflict of interest statement

The authors declare that the research was conducted in the absence of any commercial or financial relationships that could be construed as a potential conflict of interest.

## Footnotes

^1^Fungicide Resistance Action Committee (FRAC): http://www.frac.info/
