# Supplementary material for: Anilinopyrimidine Resistance in Botrytis cinerea Is Linked to Mitochondrial Function
Source: Front Microbiol. 2017 Nov 30;8:2361. doi: 10.3389/fmicb.2017.02361 (PMC5714876; doi:10.3389/fmicb.2017.02361)
Supplement: Supplementary file 1 [file Presentation1.PDF]

Supplementary Material

Anilinopyrimidine resistance in *Botrytis cinerea* is linked to mitochondrial function

Andreas Mosbach*, Dominique Edel, Andrew D. Farmer, Stephanie Widdison, Thierry Barchietto, Robert A. Dietrich, Andy Corran and Gabriel Scalliet*

***Correspondence:** andreas.mosbach@syngenta.com, gabriel.scalliet@syngenta.com

1 Supplementary Figures

- Figure S1
- Figure S2

2 Supplementary Tables

- Table S1
- Table S2
- Table S3
- Table S4
- Table S5
- Table S6


```
                      280       290       300       310       320
BcMcr1 WT (B05.10) LLGASSFKSGKGGILEQLGYRKDQIHQF.                     305
Bc-UV-CDL100-1     LLGASSFKSGKGGILEQLGIGKIRYISFNRGSGRRCRFYVIGG.      320
Bc-UV-CDL100-3     LLGLARSRVGKEASWSSWGIGKIRYISFNRGSGRRCRFYVIGG.      320
```

Figure S1 | Amino acid alignment of BcMcr1 C-terminal sequences of CDL-resistant UV mutants. Protein sequences were translated based on DNA sequencing data of isolates CDL100-1 and CDL100-3. Both showed single nucleotide deletions causing frame shift mutations, which result in longer predicted protein sequences because of the loss of the original stop codon. Residues that match the wild type reference sequence (B05.10) are shaded black.

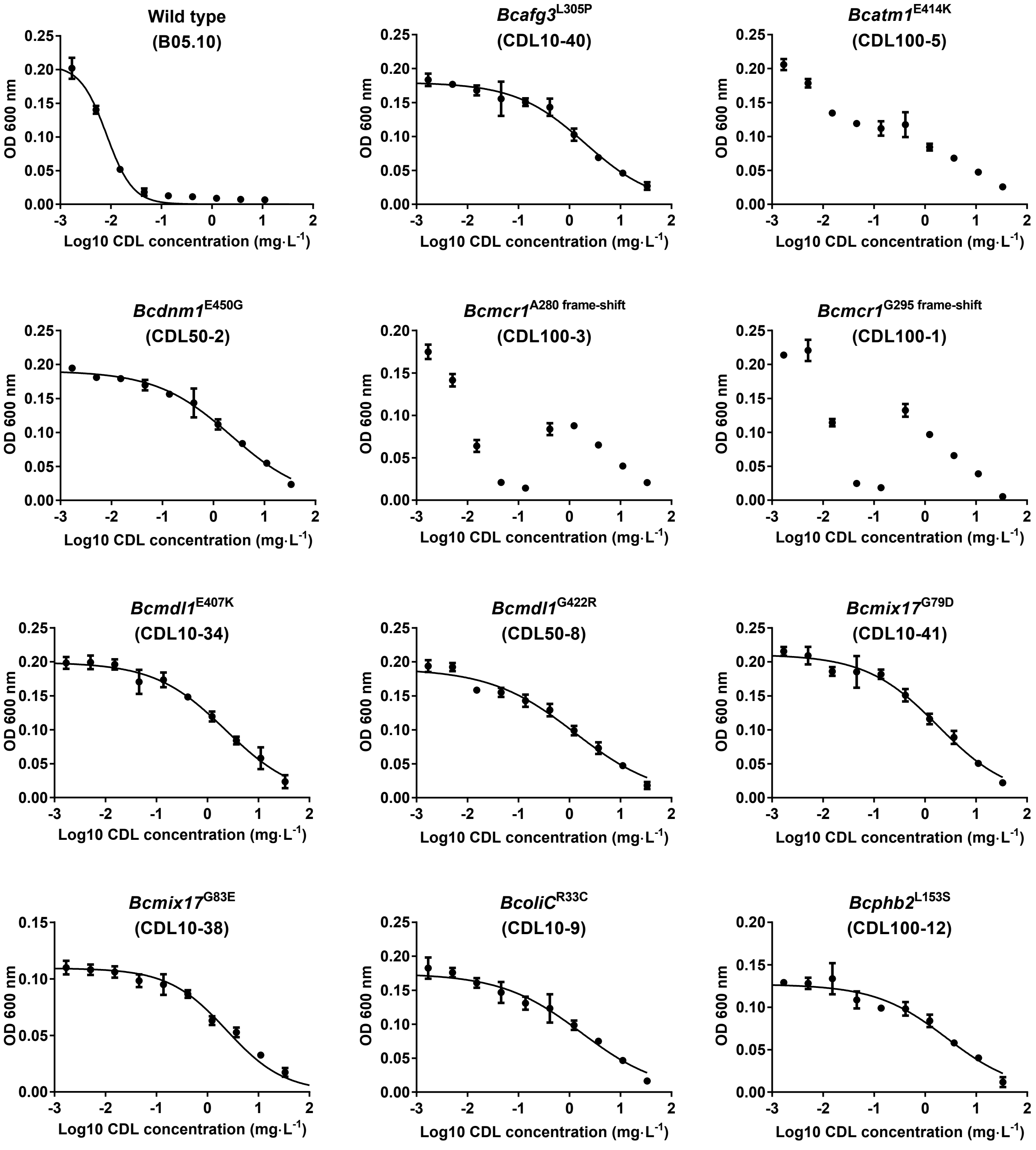

Figure S2 | CDL dose-response curves of all resistance-related mutations identified by UV screening. Representative examples per mutation are shown, with mean values of technical replications ± standard deviation. The respective isolate names are indicated in parentheses. Non-linear regression curves are included in the graphs, except for *Bcatm1*^{E414K} and the two mutants in *Bcmcr1* because of their biphasic dose-response curves.

Back to index

Table S1 | *B. cinerea* monitoring field samples collected in 2014. Sensitivity tests with CDL and FDL were conducted at BIOtransfer. Samples were classified as MDR (multidrug resistant) if the EC_{50} values towards FDL were ≥ 0.1 mg·L^{-1}. For details on pyrosequencing assays Bcmdl1-AQ-E407K, Bcpos5-SNP-L412F/V, and Bcpos5-SNP-G408R/V see Table S4. Sanger sequencing data of marked samples is listed in Table S2. Only samples are shown for which none of the pyrosequencing tests indicated a mixture of genotypes (multiple isolates per sample).

Sample information			Sensitivity tests			Pyrosequencing assays			Sanger sequencing	Final genotype
**Sample**	**Country**	**Crop**	**EC_{50} CDL (mg·L^{-1})**	**EC_{50} FDL (mg·L^{-1})**	**MDR***	**Bcmdl1-AQ-E407K**	**Bcpos5-SNP-L412F/V**	**Bcpos5-SNP-G408R/V**		
14-BC-001	Spain	Strawberry	4.4727	0.0171	no MDR	E407K	WT	WT		*Bcmdl1*^{E407K}
14-BC-002	Spain	Strawberry	0.4664	0.0155	no MDR	WT	L412F	WT		*Bcpos5*^{L412F}
14-BC-004	Spain	Strawberry	4.5834	0.0182	no MDR	WT	WT	WT	x	*Bcpos5*^{P293S}
14-BC-005	Spain	Strawberry	5.8892	0.0346	no MDR	E407K	WT	WT		*Bcmdl1*^{E407K}
14-BC-006	Spain	Strawberry	10.6892	0.0675	no MDR	E407K	WT	WT		*Bcmdl1*^{E407K}
14-BC-007	Spain	Strawberry	4.0000	0.0240	no MDR	WT	L412V	WT	x	*Bcpos5*^{L412V}
14-BC-008	Spain	Strawberry	0.0138	0.0370	no MDR	WT	WT	WT		WT
14-BC-009	Spain	Strawberry	0.0084	0.0180	no MDR	WT	WT	WT		WT
14-BC-019	Spain	Strawberry	0.6396	0.0247	no MDR	nd	WT	WT		WT
14-BC-020	Spain	Strawberry	9.1065	0.0212	no MDR	nd	WT	WT	x	*Bcmdl1*^{E407K}
14-BC-021	Spain	Strawberry	3.1004	0.0245	no MDR	WT	L412F	WT		*Bcpos5*^{L412F}
14-BC-022	Spain	Strawberry	0.0383	0.0274	no MDR	nd	WT	WT		WT
14-BC-023	Spain	Strawberry	2.1891	0.1361	MDR	WT	L412F	WT		*Bcpos5*^{L412F}
14-BC-034	Germany	Strawberry	2.0000	0.0398	no MDR	WT	L412V	WT	x	*Bcpos5*^{L412V}
14-BC-035	Germany	Strawberry	19.6139	2.5146	MDR	WT	WT	G408V	x	*Bcpos5*^{G408V}
14-BC-036	Germany	Strawberry	20.5549	1.3737	MDR	WT	WT	G408V	x	*Bcpos5*^{G408V}
14-BC-037	Germany	Strawberry	1.2663	0.0169	no MDR	WT	L412F	WT		*Bcpos5*^{L412F}
14-BC-039	Germany	Strawberry	6.7196	0.2461	MDR	WT	L412F	WT		*Bcpos5*^{L412F}
14-BC-040	Italy	Strawberry	8.5020	0.0183	no MDR	E407K	WT	WT		*Bcmdl1*^{E407K}
14-BC-041	Italy	Strawberry	4.9309	0.0245	no MDR	WT	L412F	WT		*Bcpos5*^{L412F}
14-BC-045	Sweden	Strawberry	0.0103	0.0285	no MDR	WT	WT	WT		WT
14-BC-046	Sweden	Strawberry	0.0074	0.0179	no MDR	WT	WT	WT		WT
14-BC-048	Germany	Strawberry	0.0140	0.0400	no MDR	WT	WT	WT		WT
14-BC-049	Germany	Strawberry	0.0293	0.0433	no MDR	WT	WT	WT		WT

14-BC-050	Germany	Strawberry	0.0246	0.0277	no MDR	WT	WT	WT		WT
14-BC-051	Germany	Strawberry	18.7525	2.0489	MDR	WT	WT	G408V	x	*Bcpos5*^{G408V}
14-BC-052	Germany	Strawberry	10.0000	0.5792	MDR	WT	WT	G408V	x	*Bcpos5*^{G408V}
14-BC-054	Germany	Strawberry	0.0132	0.0278	no MDR	WT	WT	WT		WT
14-BC-055	Germany	Strawberry	11.2105	1.1713	MDR	WT	L412F	WT	x	*Bcpos5*^{L412F}
14-BC-056	Germany	Strawberry	11.2310	1.7714	MDR	WT	WT	G408V	x	*Bcpos5*^{G408V}
14-BC-059	Italy	Grape	1.7598	0.0417	no MDR	WT	L412F	WT		*Bcpos5*^{L412F}
14-BC-060	Italy	Grape	1.2256	0.1147	MDR	WT	L412S	WT		*Bcpos5*^{L412S}
14-BC-061	Italy	Grape	0.5807	0.0116	no MDR	WT	L412F	WT		*Bcpos5*^{L412F}
14-BC-071	Belgium	Strawberry	0.0141	0.0327	no MDR	WT	WT	WT		WT
14-BC-073	Belgium	Strawberry	0.0119	0.0130	no MDR	E407K	WT	WT		*Bcmdl1*^{E407K}
14-BC-074	Italy	Grape	0.0156	0.0375	no MDR	E407K	WT	WT		*Bcmdl1*^{E407K}
14-BC-075	Italy	Grape	0.0118	0.0159	no MDR	WT	WT	WT		WT
14-BC-077	Italy	Grape	0.0118	0.0176	no MDR	WT	WT	WT		WT
14-BC-078	Italy	Grape	0.1450	0.0396	no MDR	WT	WT	WT		WT
14-BC-079	Italy	Grape	8.1957	0.0127	no MDR	WT	L412F	WT		*Bcpos5*^{L412F}
14-BC-083	France	Grape	0.1566	0.0120	no MDR	WT	WT	WT		WT
14-BC-084	France	Grape	0.0096	0.0280	no MDR	WT	WT	WT		WT
14-BC-085	France	Grape	0.0084	0.0107	no MDR	WT	WT	WT		WT
14-BC-086	France	Grape	0.1182	0.0132	no MDR	WT	WT	WT		WT
14-BC-087	France	Grape	0.2132	0.0383	no MDR	WT	WT	WT		WT
14-BC-088	France	Grape	0.0302	0.0113	no MDR	WT	WT	WT		WT
14-BC-089	Spain	Grape	0.0201	0.0322	no MDR	WT	WT	WT		WT
14-BC-090	Spain	Grape	0.0253	0.0247	no MDR	WT	WT	WT		WT
14-BC-091	Spain	Grape	0.0772	0.0256	no MDR	WT	WT	WT		WT
14-BC-092	Spain	Grape	0.0089	0.0383	no MDR	WT	WT	WT		WT
14-BC-093	Spain	Grape	0.1486	0.0438	no MDR	WT	WT	WT		WT
14-BC-094	Spain	Grape	0.2423	0.0133	no MDR	WT	WT	WT		WT
14-BC-095	Spain	Grape	0.0126	0.0285	no MDR	WT	WT	WT		WT
14-BC-096	Spain	Grape	0.0541	0.0169	no MDR	WT	WT	WT		WT
14-BC-097	Spain	Grape	0.0344	0.0325	no MDR	WT	WT	WT		WT
14-BC-098	Spain	Grape	0.0417	0.0208	no MDR	WT	WT	WT		WT
14-BC-099	Spain	Grape	0.0199	0.0354	no MDR	WT	WT	WT		WT

14-BC-100	Spain	Grape	0.0109	0.0282	no MDR	WT	WT	WT		WT
14-BC-101	Spain	Grape	0.0078	0.0134	no MDR	WT	WT	WT		WT
14-BC-102	Spain	Grape	0.0095	0.0107	no MDR	WT	WT	WT		WT
14-BC-103	Spain	Grape	0.0166	0.0136	no MDR	WT	WT	WT		WT
14-BC-104	Italy	Grape	0.0222	0.0242	no MDR	WT	WT	WT		WT
14-BC-105	Italy	Grape	3.7548	0.0159	no MDR	WT	WT	WT	x	*Bcpos5*^{P319A}
14-BC-106	Italy	Grape	0.0107	0.0180	no MDR	WT	WT	WT		WT
14-BC-107	Italy	Grape	5.5191	0.0423	no MDR	WT	L412F	WT		*Bcpos5*^{L412F}
14-BC-108	Italy	Grape	0.0108	0.0221	no MDR	WT	WT	WT		WT
14-BC-109	Italy	Grape	0.0930	0.0379	no MDR	WT	WT	WT		WT
14-BC-110	Italy	Grape	0.0109	0.0252	no MDR	WT	WT	WT		WT
14-BC-111	Italy	Grape	0.0142	0.0120	no MDR	WT	WT	WT		WT
14-BC-112	Italy	Grape	0.0172	0.0371	no MDR	WT	WT	WT		WT
14-BC-113	Italy	Grape	3.3175	0.0251	no MDR	WT	L412F	WT		*Bcpos5*^{L412F}
14-BC-114	Italy	Grape	18.9769	0.0466	no MDR	WT	L412F	WT		*Bcpos5*^{L412F}
14-BC-115	Italy	Grape	0.0484	0.0345	no MDR	WT	WT	WT		WT
14-BC-116	France	Grape	0.0953	0.0090	no MDR	WT	WT	WT		WT
14-BC-117	France	Grape	0.0741	0.0561	no MDR	WT	WT	WT		WT
14-BC-118	France	Grape	0.0100	0.0133	no MDR	WT	WT	WT		WT
14-BC-119	France	Grape	0.0116	0.0114	no MDR	WT	WT	WT		WT
14-BC-120	France	Grape	0.0626	0.0122	no MDR	WT	WT	WT		WT
14-BC-121	France	Grape	0.0493	0.0269	no MDR	WT	WT	WT		WT
14-BC-122	France	Grape	1.9481	0.0699	no MDR	WT	L412F	WT		*Bcpos5*^{L412F}
14-BC-123	France	Grape	12.004	0.0738	no MDR	WT	L412V	WT	x	*Bcpos5*^{L412V}
14-BC-124	France	Grape	0.0170	0.0309	no MDR	WT	WT	WT		WT
14-BC-126	France	Grape	0.0487	0.0436	no MDR	WT	WT	WT		WT
14-BC-130	France	Grape	0.1358	0.0936	no MDR	WT	WT	WT		WT
14-BC-131	France	Grape	0.8258	0.4501	MDR	WT	WT	WT		WT
14-BC-133	France	Grape	0.1569	0.0425	no MDR	WT	WT	WT		WT
14-BC-134	France	Grape	0.2742	0.0443	no MDR	WT	WT	WT		WT
14-BC-136	France	Grape	0.4505	0.0193	no MDR	WT	WT	WT		WT
14-BC-137	France	Grape	0.5704	0.0204	no MDR	WT	WT	WT		WT
14-BC-138	France	Grape	0.6489	0.4203	MDR	WT	WT	WT		WT

14-BC-139	France	Grape	0.0070	0.0100	no MDR	WT	WT	WT		WT
14-BC-140	Italy	Grape	0.0088	0.0123	no MDR	WT	WT	WT		WT
14-BC-141	Italy	Grape	0.0292	0.0369	no MDR	WT	WT	WT		WT
14-BC-142	Italy	Grape	0.0384	0.0208	no MDR	WT	WT	WT		WT
14-BC-143	Italy	Grape	0.1205	0.0302	no MDR	WT	WT	WT		WT
14-BC-144	Italy	Grape	0.0129	0.0374	no MDR	WT	WT	WT		WT
14-BC-145	Italy	Grape	0.0338	0.0165	no MDR	WT	WT	WT		WT
14-BC-146	Italy	Grape	4.2042	0.0199	no MDR	WT	L412F	WT		*Bcpos5*^{L412F}
14-BC-147	Italy	Grape	0.0400	0.0390	no MDR	WT	WT	WT		WT
14-BC-148	Italy	Grape	0.0321	0.0532	no MDR	WT	WT	WT	x	WT
14-BC-149	Italy	Grape	0.0100	0.0068	no MDR	WT	WT	WT		WT
14-BC-150	Italy	Grape	0.0309	0.0146	no MDR	WT	WT	WT		WT
14-BC-151	Italy	Grape	0.0097	0.0457	no MDR	WT	WT	WT		WT
14-BC-152	Italy	Grape	0.1791	0.0090	no MDR	WT	WT	WT		WT
14-BC-153	Italy	Grape	0.0653	0.0326	no MDR	WT	WT	WT		WT
14-BC-154	Italy	Grape	0.0897	0.0857	no MDR	WT	WT	WT		WT
14-BC-155	France	Grape	0.0300	0.0206	no MDR	WT	WT	WT		WT
14-BC-157	France	Grape	0.0594	0.0312	no MDR	WT	WT	WT		WT
14-BC-158	France	Grape	0.1292	0.0165	no MDR	WT	WT	WT		WT
14-BC-159	France	Grape	0.1992	0.0391	no MDR	WT	WT	WT		WT
14-BC-160	France	Grape	0.0190	0.0566	no MDR	WT	WT	WT		WT
14-BC-161	France	Grape	0.0245	0.0339	no MDR	WT	WT	WT		WT
14-BC-162	France	Grape	0.0111	0.0090	no MDR	WT	WT	WT		WT
14-BC-163	France	Grape	0.0140	0.0252	no MDR	WT	WT	WT		WT
14-BC-164	France	Grape	0.3545	0.0900	no MDR	WT	WT	WT		WT
14-BC-166	France	Grape	0.0105	0.0181	no MDR	WT	WT	WT		WT
14-BC-167	France	Grape	0.7581	0.2100	MDR	WT	WT	WT		WT
14-BC-168	France	Grape	0.0322	0.0299	no MDR	WT	WT	WT		WT
14-BC-169	France	Grape	0.4635	0.2354	MDR	WT	WT	WT		WT
14-BC-170	France	Grape	0.0936	0.2076	MDR	WT	WT	WT		WT
14-BC-171	France	Grape	0.0301	0.0195	no MDR	WT	WT	WT		WT
14-BC-172	France	Grape	0.9917	0.2492	MDR	WT	WT	WT	x	WT
14-BC-173	France	Grape	0.0153	0.0115	no MDR	WT	WT	WT		WT

14-BC-174	France	Grape	0.0242	0.0315	no MDR	WT	WT	WT		WT
14-BC-175	France	Grape	0.0138	0.0206	no MDR	WT	WT	WT		WT
14-BC-176	France	Grape	0.2410	0.1068	MDR	WT	WT	WT		WT
14-BC-181	Romania	Grape	3.2055	0.0378	no MDR	WT	L412F	WT		*Bcpos5*^{L412F}
14-BC-182	Romania	Grape	2.0437	0.0331	no MDR	WT	L412F	WT		*Bcpos5*^{L412F}
14-BC-183	Spain	Grape	1.7903	0.0310	no MDR	WT	WT	G408R	x	*Bcpos5*^{G408R}
14-BC-184	Spain	Grape	0.0221	0.0267	no MDR	WT	WT	WT		WT
14-BC-185	Spain	Grape	0.0399	0.0060	no MDR	WT	WT	WT		WT
14-BC-186	Spain	Grape	0.1174	0.0066	no MDR	WT	WT	WT		WT
14-BC-187	Spain	Grape	0.0354	0.0261	no MDR	WT	WT	WT		WT
14-BC-188	France	Grape	0.0117	0.0156	no MDR	WT	WT	WT		WT
14-BC-189	France	Grape	0.2282	0.0135	no MDR	WT	WT	G408R		*Bcpos5*^{G408R}
14-BC-190	France	Grape	0.0116	0.0252	no MDR	WT	WT	WT		WT
14-BC-191	France	Grape	0.0111	0.0173	no MDR	WT	WT	WT		WT
14-BC-192	France	Grape	0.0137	0.0132	no MDR	WT	WT	WT		WT
14-BC-193	France	Grape	0.0456	0.0384	no MDR	WT	WT	WT		WT
14-BC-194	France	Grape	0.3507	0.0108	no MDR	WT	WT	G408V		*Bcpos5*^{G408V}
14-BC-195	France	Grape	0.0115	0.0115	no MDR	WT	WT	WT		WT
14-BC-196	France	Grape	0.0126	0.0099	no MDR	WT	WT	WT		WT
14-BC-197	France	Grape	0.0154	0.0172	no MDR	WT	WT	WT		WT
14-BC-198	France	Grape	0.1223	0.2022	MDR	WT	WT	WT		WT
14-BC-199	France	Grape	0.0294	0.0323	no MDR	WT	WT	WT		WT
14-BC-201	France	Grape	0.3239	0.2567	MDR	WT	WT	WT		WT
14-BC-203	Italy	Grape	0.2173	0.0152	no MDR	WT	WT	WT		WT
14-BC-204	Italy	Grape	0.0369	0.0194	no MDR	WT	WT	WT		WT
14-BC-205	Italy	Grape	0.0105	0.0103	no MDR	WT	WT	WT		WT
14-BC-206	Portugal	Grape	0.0133	0.0152	no MDR	WT	WT	WT		WT
14-BC-207	Portugal	Grape	0.0175	0.0117	no MDR	WT	WT	WT		WT
14-BC-208	Portugal	Grape	0.0079	0.0123	no MDR	WT	WT	WT		WT
14-BC-209	Spain	Grape	0.0792	0.0220	no MDR	WT	WT	WT		WT
14-BC-210	Spain	Grape	0.0187	0.0124	no MDR	WT	WT	WT		WT
14-BC-211	Spain	Grape	0.0163	0.0144	no MDR	WT	WT	WT		WT
14-BC-212	Spain	Grape	0.0129	0.0117	no MDR	WT	WT	WT		WT

14-BC-213	Spain	Grape	0.0438	0.0133	no MDR	WT	WT	WT		WT
14-BC-214	Spain	Grape	0.0185	0.0173	no MDR	WT	WT	WT		WT
14-BC-215	Spain	Grape	0.0161	0.0279	no MDR	WT	WT	WT	x	WT
14-BC-216	Spain	Grape	0.0163	0.0164	no MDR	WT	WT	WT		WT
14-BC-217	France	Grape	0.8552	0.0389	no MDR	WT	L412F	WT		*Bcpos5*^{L412F}
14-BC-218	France	Grape	0.0328	0.0435	no MDR	WT	WT	WT		WT
14-BC-219	France	Grape	0.0141	0.0789	no MDR	WT	WT	WT		WT
14-BC-220	France	Grape	1.0887	0.1957	MDR	WT	L412F	WT		*Bcpos5*^{L412F}
14-BC-221	Germany	Grape	1.0996	0.1868	MDR	WT	WT	WT	x	WT
14-BC-222	Germany	Grape	12.0613	0.2254	MDR	WT	L412F	WT		*Bcpos5*^{L412F}
14-BC-223	Germany	Grape	0.1241	0.1189	MDR	WT	WT	WT		WT
14-BC-226	Germany	Grape	0.1500	0.0470	no MDR	WT	WT	WT		WT
14-BC-227	Germany	Grape	0.1367	0.1198	MDR	WT	WT	WT		WT
14-BC-228	Germany	Grape	0.3033	0.1080	MDR	WT	WT	WT		WT
14-BC-229	Germany	Grape	0.1675	0.1044	MDR	WT	WT	WT		WT
14-BC-231	Germany	Grape	0.0385	0.2390	MDR	WT	L412F	WT		*Bcpos5*^{L412F}
14-BC-232	Slovenia	Grape	0.3612	0.2532	MDR	WT	WT	WT		WT
14-BC-234	Slovenia	Grape	0.9889	0.5892	MDR	WT	WT	WT		WT
14-BC-235	Croatia	Grape	2.7206	0.1927	MDR	WT	L412F	WT		*Bcpos5*^{L412F}
14-BC-236	Croatia	Grape	2.2584	0.0248	no MDR	WT	L412F	WT		*Bcpos5*^{L412F}
14-BC-237	Croatia	Grape	0.9360	0.0892	no MDR	WT	L412F	WT		*Bcpos5*^{L412F}
14-BC-238	Croatia	Grape	0.5946	0.0208	no MDR	WT	WT	WT		WT
14-BC-239	Croatia	Grape	0.0975	0.0114	no MDR	WT	WT	WT		WT
14-BC-240	Croatia	Grape	0.4891	0.0159	no MDR	WT	WT	WT		WT
14-BC-241	France	Grape	0.0565	0.0432	no MDR	WT	WT	WT		WT
14-BC-242	France	Grape	0.0132	0.0068	no MDR	WT	WT	WT		WT
14-BC-243	France	Grape	0.0239	0.0084	no MDR	WT	WT	WT		WT
14-BC-244	France	Grape	0.0233	0.0124	no MDR	WT	WT	WT		WT
14-BC-245	France	Grape	0.0117	0.0149	no MDR	WT	WT	WT		WT
14-BC-246	France	Grape	0.0386	0.0230	no MDR	WT	WT	WT		WT
14-BC-247	France	Grape	0.0918	0.0816	no MDR	WT	WT	WT		WT
14-BC-248	France	Grape	0.0155	0.0362	no MDR	WT	WT	WT		WT
14-BC-250	Italy	Grape	0.0934	0.1214	MDR	WT	WT	WT		WT

14-BC-251	Italy	Grape	0.0128	0.0116	no MDR	WT	WT	WT		WT
14-BC-252	Italy	Grape	0.0136	0.0151	no MDR	WT	WT	WT		WT
14-BC-253	Italy	Grape	0.0107	0.0321	no MDR	WT	WT	WT		WT
14-BC-254	Italy	Grape	0.0113	0.0054	no MDR	WT	WT	WT		WT
14-BC-255	Italy	Grape	0.0253	0.0368	no MDR	WT	WT	WT		WT
14-BC-256	Italy	Grape	0.0150	0.0336	no MDR	WT	WT	WT		WT
14-BC-257	Italy	Grape	1.9415	0.0319	no MDR	WT	L412F	WT		*Bcpos5*^{L412F}
14-BC-258	Italy	Grape	0.0127	0.0251	no MDR	WT	WT	WT		WT
14-BC-260	Italy	Grape	0.0271	0.0359	no MDR	WT	WT	WT		WT
14-BC-261	Italy	Grape	0.0474	0.0403	no MDR	WT	WT	WT		WT
14-BC-262	Italy	Grape	0.0308	0.0379	no MDR	WT	WT	WT		WT
14-BC-263	Italy	Grape	0.0162	0.0238	no MDR	WT	WT	WT		WT
14-BC-264	France	Grape	0.1484	0.0126	no MDR	WT	WT	WT		WT
14-BC-265	France	Grape	0.0179	0.0120	no MDR	WT	WT	WT		WT
14-BC-266	France	Grape	0.0201	0.0112	no MDR	WT	WT	WT		WT
14-BC-267	France	Grape	0.0320	0.0446	no MDR	WT	WT	WT		WT
14-BC-268	France	Grape	0.0210	0.0118	no MDR	WT	WT	WT		WT
14-BC-269	France	Grape	0.0392	0.0119	no MDR	WT	WT	WT		WT
14-BC-270	France	Grape	0.0216	0.0129	no MDR	WT	WT	WT		WT
14-BC-271	France	Grape	0.0245	0.0392	no MDR	WT	WT	WT		WT
14-BC-272	France	Grape	0.2124	0.2545	MDR	WT	WT	WT		WT
14-BC-273	France	Grape	6.6699	0.0319	no MDR	WT	L412F	WT		*Bcpos5*^{L412F}
14-BC-277	France	Grape	1.0000	0.1726	MDR	WT	WT	G408V	x	*Bcpos5*^{G408V}
14-BC-278	France	Grape	0.0500	0.0382	no MDR	WT	WT	WT		WT
14-BC-279	France	Grape	0.0214	0.0293	no MDR	WT	WT	WT		WT
14-BC-280	France	Grape	1.0832	0.0410	no MDR	WT	L412F	WT		*Bcpos5*^{L412F}
14-BC-281	France	Grape	0.5364	0.0165	no MDR	WT	WT	WT		WT
14-BC-282	France	Grape	0.0234	0.0251	no MDR	WT	WT	WT		WT
14-BC-283	France	Grape	1.1823	0.0116	no MDR	WT	L412F	WT		*Bcpos5*^{L412F}
14-BC-284	France	Grape	0.0274	0.0196	no MDR	WT	WT	WT		WT
14-BC-285	France	Grape	1.0676	0.0106	no MDR	WT	L412F	WT		*Bcpos5*^{L412F}
14-BC-286	France	Grape	0.0659	0.0187	no MDR	WT	WT	WT		WT
14-BC-287	Germany	Grape	0.0439	0.0383	no MDR	WT	WT	WT		WT

14-BC-288	Germany	Grape	0.0837	0.1378	MDR	WT	WT	WT		WT
14-BC-289	Germany	Grape	0.4881	0.2564	MDR	WT	WT	WT		WT
14-BC-293	Switzerland	Grape	2.1859	0.0701	no MDR	WT	L412F	WT		*Bcpos5*^{L412F}
14-BC-294	Switzerland	Grape	2.2872	0.0184	no MDR	WT	L412F	WT		*Bcpos5*^{L412F}
14-BC-295	Switzerland	Grape	0.1363	0.0614	no MDR	WT	WT	WT		WT
14-BC-296	Hungary	Grape	0.0153	0.0212	no MDR	WT	WT	WT		WT
14-BC-297	Hungary	Grape	0.0389	0.0243	no MDR	WT	WT	WT		WT
14-BC-298	Hungary	Grape	0.0247	0.0116	no MDR	WT	WT	WT		WT
14-BC-299	Portugal	Grape	0.0179	0.0228	no MDR	WT	WT	WT		WT
14-BC-300	Spain	Grape	0.2271	0.0274	no MDR	WT	WT	WT		WT
14-BC-301	France	Grape	0.0255	0.0131	no MDR	WT	WT	WT		WT
14-BC-305	France	Grape	0.0964	0.1066	MDR	WT	WT	WT		WT
14-BC-306	France	Grape	0.7144	0.0067	no MDR	WT	WT	WT		WT
14-BC-307	France	Grape	0.0108	0.0463	no MDR	WT	WT	WT		WT
14-BC-308	France	Grape	0.0251	0.0360	no MDR	WT	WT	WT		WT
14-BC-309	France	Grape	0.0380	0.0139	no MDR	WT	WT	WT		WT
14-BC-310	France	Grape	4.3299	0.0425	no MDR	WT	nd	WT	x	*Bcpos5*^{L412F}
14-BC-312	France	Grape	0.0599	0.0270	no MDR	WT	WT	WT		WT
14-BC-313	France	Grape	0.0195	0.0156	no MDR	WT	WT	WT		WT
14-BC-317	Romania	Grape	0.0181	0.0160	no MDR	WT	WT	WT		WT
14-BC-318	Romania	Grape	0.0217	0.0270	no MDR	WT	WT	WT		WT
14-BC-319	Romania	Grape	0.0265	0.0082	no MDR	WT	WT	WT		WT
14-BC-320	Switzerland	Grape	0.1088	0.0766	no MDR	WT	WT	WT		WT
14-BC-321	Switzerland	Grape	0.0169	0.0257	no MDR	WT	WT	WT		WT
14-BC-322	Switzerland	Grape	0.0453	0.0347	no MDR	WT	WT	WT		WT
14-BC-324	Germany	Grape	15.8517	0.5837	MDR	WT	L412F	WT		*Bcpos5*^{L412F}
14-BC-326	Germany	Grape	0.0661	0.0525	no MDR	WT	WT	WT		WT
14-BC-327	Germany	Grape	5.2737	0.2883	MDR	WT	L412F	WT		*Bcpos5*^{L412F}
14-BC-332	Germany	Grape	9.9916	0.0176	no MDR	WT	L412F	WT		*Bcpos5*^{L412F}
14-BC-333	Germany	Grape	0.2038	0.0780	no MDR	WT	WT	WT		WT
14-BC-340	Switzerland	Grape	2.8162	0.0181	no MDR	WT	L412F	WT		*Bcpos5*^{L412F}
14-BC-341	Switzerland	Grape	0.0152	0.0235	no MDR	WT	WT	WT		WT
14-BC-342	Switzerland	Grape	0.0321	0.0383	no MDR	WT	WT	WT		WT

14-BC-343	Switzerland	Grape	0.0267	0.0318	no MDR	WT	WT	WT	WT
14-BC-344	Switzerland	Grape	0.0130	0.0193	no MDR	WT	WT	WT	WT
14-BC-345	Switzerland	Grape	0.0164	0.0211	no MDR	WT	WT	WT	WT
14-BC-346	Switzerland	Grape	0.0186	0.0058	no MDR	WT	WT	WT	WT
14-BC-348	Switzerland	Grape	0.0220	0.0390	no MDR	WT	WT	WT	WT
14-BC-349	Switzerland	Grape	0.0210	0.0739	no MDR	WT	WT	WT	WT
14-BC-350	Switzerland	Grape	0.0178	0.0141	no MDR	WT	WT	WT	WT
14-BC-351	Switzerland	Grape	0.0932	0.0275	no MDR	WT	WT	WT	WT
14-BC-352	Switzerland	Grape	0.0568	0.0277	no MDR	WT	WT	WT	WT
14-BC-353	Switzerland	Grape	0.0106	0.0082	no MDR	WT	WT	WT	WT
14-BC-354	Switzerland	Grape	0.0286	0.0184	no MDR	WT	WT	WT	WT
14-BC-355	Switzerland	Grape	0.0249	0.0099	no MDR	WT	WT	WT	WT
14-BC-357	France	Grape	0.0415	0.0119	no MDR	WT	WT	WT	WT
14-BC-358	France	Grape	0.0620	0.0752	no MDR	WT	WT	WT	WT
14-BC-360	France	Grape	0.0128	0.0362	no MDR	WT	WT	WT	WT
14-BC-361	Spain	Grape	0.0181	0.0108	no MDR	WT	WT	WT	WT
14-BC-362	Spain	Grape	0.0145	0.0234	no MDR	WT	WT	WT	WT
14-BC-364	France	Grape	0.0084	0.0452	no MDR	WT	WT	WT	WT
14-BC-365	France	Grape	0.0091	0.0195	no MDR	WT	WT	WT	WT
14-BC-366	France	Grape	0.0194	0.0173	no MDR	WT	WT	WT	WT
14-BC-367	France	Grape	4.9556	0.2404	MDR	WT	L412F	WT	*Bcpos5*^{L412F}
14-BC-368	France	Grape	0.6494	0.0114	no MDR	WT	L412F	WT	*Bcpos5*^{L412F}
14-BC-369	France	Grape	2.1716	0.4754	MDR	WT	L412F	WT	*Bcpos5*^{L412F}
14-BC-371	Germany	Grape	0.7057	0.2484	MDR	WT	WT	WT	WT
14-BC-372	Germany	Grape	1.1979	0.1743	MDR	WT	L412F	WT	*Bcpos5*^{L412F}
14-BC-374	Germany	Grape	3.0197	0.5552	MDR	WT	L412F	WT	*Bcpos5*^{L412F}
14-BC-377	Germany	Grape	0.4571	0.0855	no MDR	WT	L412V	WT	*Bcpos5*^{L412V}
14-BC-378	Germany	Grape	1.0564	0.0520	no MDR	WT	L412F	WT	*Bcpos5*^{L412F}
14-BC-379	Germany	Grape	3.7185	0.3757	MDR	WT	L412F	WT	*Bcpos5*^{L412F}
14-BC-380	Germany	Grape	3.2203	0.1379	MDR	WT	L412F	WT	*Bcpos5*^{L412F}
14-BC-381	Germany	Grape	14.4484	0.1454	MDR	WT	L412F	WT	*Bcpos5*^{L412F}
14-BC-383	France	Grape	2.7882	0.0111	no MDR	WT	L412F	WT	*Bcpos5*^{L412F}
14-BC-384	France	Grape	0.0143	0.0313	no MDR	WT	WT	WT	WT

14-BC-385	France	Grape	0.0141	0.0151	no MDR	WT	WT	WT		WT
14-BC-386	France	Grape	0.5738	0.0529	no MDR	WT	WT	WT		WT
14-BC-390	Italy	Strawberry	2.9220	0.0189	no MDR	E407K	WT	WT		*Bcmdl1*^{E407K}
14-BC-391	Spain	Grape	0.0107	0.0140	no MDR	WT	WT	WT		WT
14-BC-392	Spain	Grape	0.0494	0.1021	MDR	WT	WT	WT		WT
14-BC-393	Spain	Grape	0.0112	0.0253	no MDR	WT	WT	WT		WT
14-BC-394	Germany	Grape	3.3180	0.1490	MDR	WT	L412F	WT		*Bcpos5*^{L412F}
14-BC-395	Germany	Grape	0.5828	0.1763	MDR	WT	WT	WT		WT
14-BC-396	Germany	Grape	0.2176	0.4620	MDR	WT	WT	WT		WT
14-BC-397	Germany	Grape	0.1304	0.3214	MDR	WT	WT	WT		WT
14-BC-398	Germany	Grape	0.1265	0.0709	no MDR	WT	WT	WT		WT
14-BC-399	Germany	Grape	0.1301	0.1783	MDR	WT	WT	WT		WT
14-BC-400	Germany	Grape	9.5764	0.1853	MDR	WT	L412F	WT		*Bcpos5*^{L412F}
14-BC-401	France	Grape	0.0241	0.0187	no MDR	WT	WT	WT		WT
14-BC-402	France	Strawberry	10.2385	0.0213	no MDR	WT	L412F	WT		*Bcpos5*^{L412F}
14-BC-412	Germany	Grape	2.1908	0.0138	no MDR	WT	L412F	WT		*Bcpos5*^{L412F}
14-BC-413	Germany	Grape	0.0470	0.0473	no MDR	WT	WT	WT		WT
14-BC-414	Germany	Grape	0.4390	0.0842	no MDR	WT	WT	WT		WT
14-BC-415	Spain	Grape	3.6025	0.0180	no MDR	WT	WT	WT	x	*Bcpos5*^{G408R}
14-BC-416	Spain	Grape	0.0140	0.0216	no MDR	WT	WT	WT		WT

Table S2 | *B. cinerea* field samples referred to in this study with Sanger sequencing data in CDL resistance-related genes. Blank fields: Not sequenced. **-**: No non-synonymous SNP found (synonymous SNPs not shown). ^{S}Single spore isolate of corresponding field sample.

Isolate name	Crop	CDL res. phenotype	**Sanger sequencing results in CDL resistance-related genes except *Bcmcr1*, non-synonymous SNPs only**								Final genotype
			Bcmdl1	***Bcpos5***	***Bcafg3***	***Bcatm1***	***Bcdnm1***	***Bcmix17***	***BcoliC***	***Bcphb2***	
09-BC-029	Strawberry	Ani^{R1}	S29P, K790R	L412V	-	T62A, K700Q	-	-	-	-	*Bcpos5*^{L412V}
10-BC-041^{S}	Strawberry	Ani^{R1}	S29P, S466R, D780N, K790R		-	-	-	R6K	-	-	*Bcmdl1*^{S466R}
10-BC-187	Grape	Ani^{R1}	S29P, T66A, D780N, K790R	V273I	-	T62A, K700Q	-	-	-	-	*Bcpos5*^{V273I}
10-BC-228	Grape	Ani^{R1}	S29P, K790R	G408V	-	R41L, T62A	-	-	-	-	*Bcpos5*^{G408V}
10-BC-258	Grape	Ani^{R1}	S29P, K790R	L412F	-	T62A,	-	-	-	-	*Bcpos5*^{L412F}
11-BC-060^{S}	Strawberry	Ani^{R1}	S29P, K790R	G408V	-	-	-	-	-	-	*Bcpos5*^{G408V}
11-BC-093	Strawberry	Ani^{R1}	S29P, K790R	G408V	-	-	-	M135V	-	-	*Bcpos5*^{G408V}
12-BC-024	Strawberry	Ani^{R1}	S29P, K790R	L412V	-	T62A, P71S, T453S, K700Q	Q94K	R6K	-	-	*Bcpos5*^{L412V}
14-BC-004	Strawberry	Ani^{R1}	S29P	P293S							*Bcpos5*^{P293S}
14-BC-007^{S}	Strawberry	Ani^{R1}	S29P, K790R	L412V							*Bcpos5*^{L412V}
14-BC-020^{S}	Strawberry	Ani^{R1}	S29P, E407K, D780N, K790R	-							*Bcmdl1*^{E407K}
14-BC-025	Tomato	Ani^{R1}	S29P,D780N, K790R	-	-	K700Q	-	-	-	-	Unexplained
14-BC-029	Tomato	Ani^{R1}	S29P, D780N, K790R	-	-	K700Q	-	-	-	-	Unexplained
14-BC-030	Tomato	Ani^{R1}	S29P, D780N, K790R	-							Unexplained
14-BC-031	Tomato	Ani^{R1}	S29P, G40A, K790R	L412V							*Bcpos5*^{L412V}
14-BC-032	Tomato	Ani^{R1}	S29P, G40A, K790R	L412V							*Bcpos5*^{L412V}
14-BC-033	Tomato	Ani^{R1}	S29P, G40A, K790R	L412V							*Bcpos5*^{L412V}
14-BC-034	Strawberry	Ani^{R1}	S29P, K790R	L412V							*Bcpos5*^{L412V}
14-BC-035	Strawberry	Ani^{R1}	S29P, K790R	H186Y, G408V							*Bcpos5*^{G408V}

14-BC-036	Strawberry	Ani^{R1}	S29P, K790R	H186Y, G408V							*Bcpos5*^{G408V}
14-BC-038	Strawberry	Ani^{R1}	S29P, K790R	H186Y, G408V							*Bcpos5*^{G408V}
14-BC-051	Strawberry	Ani^{R1}	S29P, K790R	H186Y, G408V							*Bcpos5*^{G408V}
14-BC-052	Strawberry	Ani^{R1}	S29P, K790R	H186Y, G408V							*Bcpos5*^{G408V}
14-BC-056	Strawberry	Ani^{R1}	S29P, K790R	G408V							*Bcpos5*^{G408V}
14-BC-066	Tomato	Ani^{R1}	S29P,D780N, K790R	-							Unexplained
14-BC-105	Grape	Ani^{R1}	S29P, T66A, D780N, K790R	P319A	-	-	-	M135V	-	-	*Bcpos5*^{P319A}
14-BC-123	Grape	Ani^{R1}	S29P, T66A, D780N, K790R	L412V							*Bcpos5*^{L412V}
14-BC-148	Grape	Sensitive	S29P, K790R	-							
14-BC-172	Grape	Ani^{R2}	S29P, K790R	-	-	T453S, K700Q	-	-	-	-	MDR1 (Ani^{R2})
14-BC-183^{s}	Grape	Ani^{R1}	S29P, K790R	G408R	-	T453S, K700Q	-	-	-	-	*Bcpos5*^{G408R}
14-BC-215	Grape	Sensitive	S29P, K790R	-	-	T453S, K700Q	-	-	-	-	
14-BC-310	Grape	Ani^{R1}	S29P, S610P, K790R	L412F	-	K700Q	-	-	-	-	*Bcpos5*^{L412F}
14-BC-328	Tomato	Ani^{R1}	S29P, D780N, K790R	L412V							*Bcpos5*^{L412V}
14-BC-370	Grape	Ani^{R1}	S29P, K790R	G408V							*Bcpos5*^{G408V}
14-BC-375	Tomato	Ani^{R1}	S29P, D780N, K790R	L412V							*Bcpos5*^{L412V}
14-BC-376	Tomato	Ani^{R1}	S29P, D780N, K790R	L412V							*Bcpos5*^{L412V}
14-BC-415	Grape	Ani^{R1}	S29P, K790R	G408R	-	-	-	-	-	-	*Bcpos5*^{G408R}
BAR633	Grape	Ani^{R1}	S29P, E407K, K790R	-	-	T62A, P71S, T453S, K700Q	-	-	-	-	*Bcmdl1*^{E407K}

Table S3 | PCR primers used to amplify sequences for reverse genetic experiments (transformation of resistance-conferring alleles into the sensitive wild type B05.10) and for Sanger sequencing. *Primer Tm shown for Phusion Hot Start II High-Fidelity DNA Polymerase.

Gene ID or locus	Purpose	Primer name	Oligonucleotide sequence (5'-3' orientation)	Tm (°C)
Bcafg3 (Bcin03g02170)	Reverse genetic validation, 5' part of CDS including SNP	Bcafg3_HR_for	AGGCATCATAATGATTTGCCTCCCAC	72.0*
		Bcafg3_HR_rev	GGCGAAAAGATCTCGAACTCTCGATG	72.4*
	PCR for Sanger sequencing	Bcafg3_S_for	AGGCATCATAATGATTTGCCTCCCAC	72.0*
		Bcafg3_S_rev	CTTCCTTTTCTCTTCATTGATTCATCGAC	69.2*
Bcatm1 (Bcin15g00830)	Reverse genetic validation, complete CDS including SNP	Bcatm1_HR_for	TCAGCCTCACTATCTTTGCGGG	69.6*
		Bcatm1_HR_rev	CCCATCACTACATGATCAAAGCGTTGTC	72.5*
	PCR for Sanger sequencing (for2/rev2 for validation of SNP in transformants)	Bcatm1_S_for	GATATTCCTCTCGATTGTGCCACTTTC	69.3*
		Bcatm1_S_for2	ATCAGCAAGCCATCCTTCCTATCC	69.3*
		Bcatm1_S_rev	ACTTTGAAAGTTGATGCTTGGTGGTCTG	71.4*
		Bcatm1_S_rev2	TGGCTCGGAAACTGGCATGG	72.8*
Bcdnm1 (Bcin02g02630)	Reverse genetic validation, middle part of CDS including SNP	Bcdnm1_HR_for	GGGATTTATCGGTGTCGTCAACAGATCACAG	75.7*
		Bcdnm1_HR_rev	AAGTTCGTAATCATCCTCACGAGCTTGTTGGGC	77.9*
	PCR for Sanger sequencing	Bcdnm1_S_for	CTTGATATACCACAGAGCCTCCTCGAATC	70.8*
		Bcdnm1_S_rev	CATATTCCATAAGTAGTGTGGACAATGCAGAG	70.0*
Bcmcr1 (Bcin10g00060)	Reverse genetic validation, complete CDS including 1 bp deletion	Bcmcr1_HR_for	TTTGCACATTTCACAGTTCAACGGCG	75.6*
		Bcmcr1_HR_rev	GGAGTAACTCCCGGGCTGTGTTATTTC	71.4*
	PCR for Sanger sequencing (validation of transformants)	Bcmcr1_S_for	TCTCTCGGACTTAGTGTGCGCG	71.0*
		Bcmcr1_S_rev	TGCTGTCGTACACCTACCCATGTCTAG	69.8*
Bcmdl1 (Bcin16g00820)	Reverse genetic validation, middle part of CDS including SNPs	Bcmdl1_HR_for	GACTTGGCTACTAAAGATCCCTCGGAAG	70.1*
		Bcmdl1_HR_rev	CCGATAATTGGGCACCACGTGC	74.1*
	Reverse genetic validation, 3' part of CDS including SNPs	Bcmdl1_HR_for2	GGAAAGCGAGAAGCATTGATTAGTGC	70.3*
		Bcmdl1_HR_rev2	TATCGCTTGTCGCTCCGAAG	67.8*
	PCR for Sanger sequencing (rev2 for validation of SNP in transformants)	Bcmdl1_S_for	TGTTGCCCGTGTAGAAGAGAATCCTTC	71.6*
		Bcmdl1_S_rev	CTATAACAAGCCGGTATGAGACTCTCGC	70.0*
		Bcmdl1_S_rev2	CGCAGCTCATGGACTCAATCTTTC	70.1*
Bcmix17 (Bcin01g01830)	Reverse genetic validation, complete CDS including SNP	Bcmix17_HR_for	GGGATGATACCATGATTTGTCACGGC	73.3*
		Bcmix17_HR_rev	AGCTATTAGCAACACATCTCGCTCTCGAGG	73.6*
	PCR for Sanger sequencing (rev2 for validation of SNP in transformants)	Bcmix17_S_for	GGGATGATACCATGATTTGTCACGGC	73.3*
		Bcmix17_S_rev	AGCTATTAGCAACACATCTCGCTCTCGAGG	73.6*
		Bcmix17_S_rev2	ACCACAACGCTTGGTAGGGAATTCAG	72.2*
BcoliC (Bcin10g01500)	Reverse genetic validation, complete CDS including SNP	BcoliC_HR_for	TCTACACCTGTACTCTCTATCCGGGC	68.3*
		BcoliC_HR_rev	TTATACACACCCCCTCCCCATTTC	69.4*
	PCR for Sanger sequencing (for2 for validation of SNP in transformants)	BcoliC_S_for	TCTACACCTGTACTCTCTATCCGGGC	68.3*
		BcoliC_S_for2	CGCACAATAGCTGGCGTAAGAAGTCTC	71.9*
		BcoliC_S_rev	CATACATACAACCTGCTGGCGTTTGG	72.3*
Bcpet9 (Bcin03g01010)	Reverse genetic validation attempt, 3' part of CDS including SNP	Bcpet9_HR_for	GCACAATTTCCATTCCTTCGGGTG	72.8*
		Bcpet9_HR_rev	CCTTGCCTCCAACTTTCCCTAGTCTTAG	70.0*

Bcphb2 (Bcin07g01710)	Reverse genetic validation, 3’ part of CDS including SNP	Bcphb2_HR_for	GTCGAACAATCCGCAAGATGTTTTCAAGAGGCTGC	80.9*
		Bcphb2_HR_rev	GCTCAGGTTCCGCTGCACATCTTGCCCAC	82.4*
	PCR for Sanger sequencing	Bcphb2_S_for	ATCGCGCTTCACGACTTTCACTCTG	73.2*
		Bcphb2_S_rev	GCTCAGGTTCCGCTGCACATCTTGCCCAC	82.4*
Bcpos5 (Bcin10g02880)	Reverse genetic validation, complete (for1) or 3’ part of CDS (for2) including SNPs	Bcpos5_HR_for1	TGGAACTTGCGGGAAGCTTG	69.8*
		Bcpos5_HR_for2	GGTGTTGCCCAGGTTTCGCTCCATG	78.0*
		Bcpos5_HR_rev	GGAATCAACCGAAGGTGCCG	71.1*
	PCR for Sanger sequencing (rev2 for validation of SNP in transformants)	Bcpos5_S_for	GATTAGACTAGGCAGATTTGTCGTGTGCTC	70.9*
		Bcpos5_S_rev	GCTGGTCCGGGTTGTAGGCAACGTG	77.9*
		Bcpos5_S_rev2	GAAATGCGCCAGCAACGTGG	73.2*
Bcin10-1 (Chr10: 1074560-1086404)	Reverse genetic approach to identify the resistance-conferring locus within the Ani^{R1} (*Bcpos5*) mapping window on the *B. cinerea* chromosome 10 (cross 09Bc11 x BAR633), names of transformed PCR fragments (see: Figure 2) and positions on chromosome 10 are indicated	Bcin10-1_HR_for	CGATTTCACTCGCACCTTCTTCAGCC	64.7
		Bcin10-1_HR_rev	ATTGCTTAAGAAGCCAGAGCCAAGACTGCG	67.5
Bcin10-1-1 (Chr10: 1074560-1078154)		Bcin10-1-1_HR_for	CGATTTCACTCGCACCTTCTTCAGCC	64.7
		Bcin10-1-1_HR_rev	TGTTCCATCTCCACCGAGTGTTGTTACC	63.8
Bcin10-1-2 (Chr10: 1077456-1081069)		Bcin10-1-2_HR_for	GGACAAGGCAGGGAAGTTCTCTGGC	64.0
		Bcin10-1-2_HR_rev	TTGGGTATAGATCCAACAACCGGGTG	62.5
Bcin10-1-1/2 (Chr10: 1074560-1081069)		Bcin10-1-1/2_HR_for	CGATTTCACTCGCACCTTCTTCAGCC	64.7
		Bcin10-1-1/2_HR_rev	TTGGGTATAGATCCAACAACCGGGTG	62.5
Bcin10-1-3 (Chr10: 1080060-1083681)		Bcin10-1-3_HR_for	GTCACACCAAATGGAACAACGTGACG	63.0
		Bcin10-1-3_HR_rev	CACAATTCGTCTTCGGATCCCATCTC	62.6
Bcin10-1-4 (Chr10: 1082772-1086404)		Bcin10-1-4_HR_for	CGCGGTAATCAAGTTTCATGCGATGGATCG	70.7
		Bcin10-1-4_HR_rev	ATTGCTTAAGAAGCCAGAGCCAAGACTGCG	67.5
Bcin10-2 (Chr10: 1084891-1096423)		Bcin10-2_HR_for	TGCGTCCAGCCAGGTTCAATCTCG	65.9
		Bcin10-2_HR_rev	GTTTCTCGGTGCGCCACGAGTAGACTCAGC	70.3
Bcin10-3 (Chr10: 1095738-1106866)		Bcin10-3_HR_for	CCAGAGCCACCAGATCCATCATCATCTTTC	66.9
		Bcin10-3_HR_rev	TCGCCTTATCATCGTTGCAGAGTGAACACG	68.8
Bcin10-4 (Chr10: 1106160-1117811)		Bcin10-4_HR_for	TAATTGCGGTTCCGAGGGTGTCTCAGGGC	71.6
		Bcin10-4_HR_rev	GCACTAGCTGGAAGATCGAGGATATGAACC	64.0
Bcin10-5 (Chr10: 1116232-1127335)		Bcin10-5_HR_for	ATGCTGTCACCAAGAACTCCGGGCC	66.7
		Bcin10-5_HR_rev	GGCTCCATGATCTGAAACTCCATTCTCG	64.6
Bcin10-6 (Chr10: 1173007-1184070)		Bcin10-6_HR_for	CATGTCAGCTTCGAGTTCTGATGTCAGTGG	65.6
		Bcin10-6_HR_rev	GCGATGGTTGTAGCTCGAGACTAGACACC	64.1
Bcin10-7 (Chr10: 1182521-1193596)		Bcin10-7_HR_for	TGGAGCTTGAGAAATTTAGCGCGAGTCTCG	68.3
		Bcin10-7_HR_rev	GGGTCGAGGGCGTTCACAATAGTGAG	64.5
Bcin10-8 (Chr10: 1193062-1204187)		Bcin10-8_HR_for	GCTAGGTAGATTCCGTGAATCAGAGTTGCG	64.7
		Bcin10-8_HR_rev	CTTGACCCTATCAAGCCACTGAAGATCG	63.0
Bcin10-9 (Chr10: 1202887-1213933)		Bcin10-9_HR_for	GAACAGACTCTTAGAGAAAGCTCCGCTTCC	63.1
		Bcin10-9_HR_rev	GCATCATCGCGTCGCACATTATCG	64.9
Bcin10-10 (Chr10: 1212061-1223085)		Bcin10-10_HR_for	GACGTCCTGCAGCCAAACTTTGTGC	64.6
		Bcin10-10_HR_rev	TGGATGGGGATGACTCCGCAGAAC	64.8

Table S4 | Pyrosequencing primers and assay conditions. AQ: Allele quantification assay. SNP: SNP genotyping assay. [B]Biotinylated primer. [F/R]Forward / Reverse sequencing primer.

Gene ID	Assay name	Mutant allele	Primer name	Sequence (5'-3' orientation)	Dispensation order
Bcmdl1	Bcmdl1-AQ-E407K	E407K (GAA▶AAA)	Bcmdl1-AQ-407_for[B]	GGAGAGACTCGGAAATGTTCG	GCTACGCTC
			Bcmdl1-AQ-407_rev	TAAAAGCTGGAGACACCAAACA	
			Bcmdl1-AQ-407_seq[R]	TTGCACTAATCAATGCTT	
Bcpos5	Bcpos5-AQ-L412F	L412F (TTG▶TTC/T)	Bcpos5-AQ-412_for	GGGGCGTTCCTTGTGTTATG	ATGCATGAGT
			Bcpos5-AQ-412_rev[B]	CGCCAAAAGGATGGTTGAA	
			Bcpos5-AQ-412_seq[F]	GAGGCTTGAATGGGT	
	Bcpos5-SNP-G408R/V	G408R/V (GGC▶CGC) (GGC▶GTC)	Bcpos5-SNP-408_for	GTGTTATGAGAGGGGCCAAAAGTG	ACGTCATGATGTG
			Bcpos5-SNP-408_rev[B]	TTCGCCAAAAGGATGGTTGAA	
			Bcpos5-SNP-408_seq[F]	GACGGTTGGGTTGGA	
	Bcpos5-SNP-L412F/V	L412F/V (TTG▶TTC/T) (TTG▶GTG)	Bcpos5-SNP-412_for	TTATGAGAGGGGCCAAAAGTG	CGTGACTGAG
			Bcpos5-SNP-412_rev[B]	CGCCAAAAGGATGGTTGAA	
			Bcpos5-SNP-412_seq[F]	TGGAGGCTTGAATGG	

Table S5 | Conditions used to select CDL-resistant mutants of *B. cinerea* reference strain B05.10, and outcome of the UV screening. Non-UV-treated controls were done on a limited number of plates (1 or 2 per condition). A total of 5 spontaneous CDL-resistant colonies (3 at 10 mg·L^{-1} and 2 at 20 mg·L^{-1}) were obtained in the second round of mutagenesis only but were not analyzed further. *Isolates were phenotypically tested and only those showing stable resistance were used for further experiments. Selection criteria used were: Growth on new selective Vogel's agar plates with CDL (10 or 20 mg·L^{-1} depending on the origin of the isolates) for the 1st and > 50 % growth in a liquid culture test relative to the untreated control at a CDL concentration of 0.05 mg·L^{-1} in liquid Vogel's minimal medium with sucrose in the 2nd round of UV mutagenesis. Isolate names referred to in the text comprise the selective concentration they were isolated from (for example: CDL50-2 is isolate number 2 selected on CDL at 50 mg·L^{-1}).

UV screening	CDL concentration (mg·L^{-1})	Conidia per plate	Number of plates	Number of isolates picked in total	Number of isolates showing stable resistance*
1st round	10	1.10^6	25	9	8
		5.10^6	5	12	6
	20	1.10^6	25	2	2
		5.10^6	5	4	4
2nd round	10	1.10^6	10	5	0
		2.10^7	10	18	11
	20	1.10^6	10	5	0
		2.10^7	10	4	1
	50	1.10^6	20	8	5
		2.10^7	20	27	16
	100	1.10^6	20	-	-
		2.10^7	20	20	13

Table S6 | Non-synonymous high-confidence SNPs identified by whole-genome sequencing of CDL-resistant UV mutants. Results were filtered for high confidence SNPs, and mutant frequencies of ≥ 0.7 were highlighted in red. RefAA / VarAA: Reference / variant amino acid. B05.10: Reference strain. Names of UV mutants CDL10-9 to CDL100-12 are abbreviated. *Mutation resulting in stop codon or frame shift. †Filters applied: Frequencies of variants ≥ 0.7 in UV isolates and ≤ 0.1 in the reference strain, only results shown with coverage (sequencing reads) of ≥ 10 in B05.10.

Chrom.	Position	SNP	Gene	RefAA	VarAA	SNP frequency in sample (UniqAlns / UniqCoverage per position)													
						B05.10	10-9	10-11	10-12	10-34	10-40	10-41	20-3	50-2	50-8	100-3	100-5	100-11	100-12
BCIN10	575989	C►T	Bcin10g01500	R	C	0.0	1.0	0.0	0.0	0.0	0.0	0.0	1.0	0.0	0.0	0.0	0.0	0.0	0.0
BCIN01	710028	C►T	Bcin01g01830	G	D	0.0	0.0	0.0	1.0	0.0	0.0	1.0	0.0	0.0	0.0	0.0	0.0	0.0	0.0
BCIN08	493068	C►T	Bcin08g01240	E	K	0.0	0.0	0.0	0.0	1.0	0.0	0.0	0.0	0.0	0.0	0.0	0.0	0.0	0.0
BCIN10	1655975	C►T	Bcin10g04290	G	R	0.0	0.0	0.0	0.0	0.8	0.0	0.0	0.0	0.0	0.0	0.0	0.0	0.0	0.0
BCIN11	340654	G►A	Bcin11g00970	P	S	0.0	0.0	0.0	0.0	1.0	0.0	0.0	0.0	0.0	0.0	0.0	0.0	0.0	0.0
BCIN16	201007	G►A	Bcin16g00360	P	S	0.0	0.0	0.0	0.0	1.0	0.0	0.0	0.0	0.0	0.0	0.0	0.0	0.0	0.0
BCIN16	361160	C►T	Bcin16g00820	E	K	0.0	0.0	0.0	0.0	1.0	0.0	0.0	0.0	0.0	0.0	0.0	0.0	0.0	0.0
BCIN02	2494619	C►T	Bcin02g07040	D	N	0.0	0.0	0.0	0.0	0.0	0.8	0.0	0.0	0.0	0.0	0.0	0.0	0.0	0.0
BCIN03	726663	T►C	Bcin03g02170	L	P	0.0	0.0	0.0	0.0	0.0	1.0	0.0	0.0	0.0	0.0	0.0	0.0	0.0	0.0
BCIN02	1187669	C►T	Bcin02g03300	G	S	0.0	0.0	0.0	0.0	0.0	0.0	0.7	0.0	0.0	0.0	0.0	0.0	0.0	0.0
BCIN03	949949	G►A	Bcin03g02860	A	V	0.0	0.0	0.0	0.0	0.0	0.0	0.0	1.0	0.0	0.0	0.0	0.0	0.0	0.0
BCIN09	942270	G►A	Bcin09g02620	T	I	0.0	0.0	0.0	0.0	0.0	0.0	0.0	1.0	0.0	0.0	0.0	0.0	0.0	0.0
BCIN01	3651624	G►T	Bcin01g10470	A	S	0.0	0.0	0.0	0.0	0.0	0.0	0.0	0.0	1.0	0.0	0.0	0.0	0.0	0.0
BCIN02	944524	T►C	Bcin02g02630	E	G	0.0	0.0	0.0	0.0	0.0	0.0	0.0	0.0	1.0	0.0	0.0	0.0	0.0	0.0
BCIN02	786471	G►A	Bcin02g02120	P	L	0.0	0.0	0.0	0.0	0.0	0.0	0.0	0.0	0.0	1.0	0.0	0.0	0.0	0.0
BCIN16	361115	C►G	Bcin16g00820	G	R	0.0	0.0	0.0	0.0	0.0	0.0	0.0	0.0	0.0	1.0	0.0	0.0	0.0	0.0
BCIN10	23458	C► -	Bcin10g00060	A	(L)*	0.0	0.0	0.0	0.0	0.0	0.0	0.0	0.0	0.0	0.0	0.9	0.0	0.0	0.0
BCIN01	1098094	C►T	Bcin01g03010	G	E	0.0	0.0	0.0	0.0	0.0	0.0	0.0	0.0	0.0	0.0	0.0	1.0	0.0	0.0
BCIN07	669430	G►T	Bcin07g01780	H	N	0.0	0.0	0.0	0.0	0.0	0.0	0.0	0.0	0.0	0.0	0.0	1.0	0.0	0.0
BCIN08	1125993	G►A	Bcin08g02970	W	*	0.0	0.0	0.0	0.0	0.0	0.0	0.0	0.0	0.0	0.0	0.0	1.0	0.0	0.0
BCIN14	983339	C►T	Bcin14g02500	R	*	0.0	0.0	0.0	0.0	0.0	0.0	0.0	0.0	0.0	0.0	0.0	1.0	0.0	0.0
BCIN15	325418	G►A	Bcin15g00830	E	K	0.0	0.0	0.0	0.0	0.0	0.0	0.0	0.0	0.0	0.0	0.0	1.0	0.8	0.0
BCIN02	3055316	G►A	Bcin02g08600	Q	*	0.0	0.0	0.0	0.0	0.0	0.0	0.0	0.0	0.0	0.0	0.0	0.0	0.8	0.0
BCIN07	625540	T►C	Bcin07g01710	L	S	0.0	0.0	0.0	0.0	0.0	0.0	0.0	0.0	0.0	0.0	0.0	0.0	0.0	1.0
BCIN09	1111818	C►T	Bcin09g03100	G	R	0.0	0.0	0.0	0.0	0.0	0.0	0.0	0.0	0.0	0.0	0.0	0.0	0.0	1.0
Total number of high-confidence SNPs / indels per UV isolate†						-	10	3	4	8	3	3	17	12	11	2	9	5	25
